# Transformation Equivalence of Neural Networks

**DOI:** 10.3390/e28090946

**Published:** 2026-08-23

**Authors:** Masaki Kobayashi

**Affiliations:** Graduate School of Informatics, Nagoya University, Furo-cho, Chikusa-ku, Nagoya 4648601, Japan; masaki@hdnnlab.com

**Keywords:** complex-valued neural networks, quaternion, multilayer perceptron, I/O-equivalence, singularity

## Abstract

Multilayer perceptrons (MLPs) are considered as a singular model of learning machines. Singularities cause local minima and plateaus in the learning process. I/O-equivalence, where two different MLPs are regarded as the same multivariable function, is an important concept for understanding singularities in neural networks. In this paper, I/O-equivalence is extended to T-equivalence, which is a concept where two MLPs yield the same results through a transformation of input and output. We provide constructive families and procedures for obtaining T-equivalent networks of real-, complex-, and quaternion-valued neural networks. In particular, T-equivalence of quaternion-valued neural networks is much more complicated than that of the others.

## 1. Introduction

Multilayer perceptrons (MLPs) have been studied by many researchers for several decades. The equivalence of MLPs is an important problem for understanding information processing by MLPs. MLPs are considered multivariable functions. If two different MLPs are regarded as the same multivariable function, they are referred to as I/O-equivalent. Sussmann determined the I/O-equivalence of real-valued MLPs (RVMLPs) [1]. Moreover, he provided methods to reduce the number of hidden neurons, which can make any RVMLPs minimal. Only minimal MLPs are considered in this paper. Fukumizu and Amari analyzed local minima and plateaus in the learning process of RVMLPs using the results of Sussmann [2]. Amari proposed the use of learning algorithms, such as the natural gradient learning algorithm, to avoid singular points based on information geometry [3,4]. Complex-valued MLPs (CVMLPs) have been proposed by several researchers [5,6,7]. I/O-equivalence is also an important problem for CVMLPs. The I/O-equivalence of CVMLPs whose hidden neurons have no bias terms was determined by Nitta [8]. However, for CVMLPs with bias terms, it is much more difficult [9,10,11]. Nitta investigated local minima and plateaus in the learning process of CVMLPs based on I/O-equivalence [12]. He also provided a natural gradient learning algorithm for CVMLPs [13]. CVMLPs have been extended to quaternion-valued MLPs (QVMLPs), and the I/O-equivalence of QVMLPs has also been investigated [14,15].

I/O-equivalent MLPs are considered to provide the same information processing. If we can get the same results from two MLPs through the transformation of input and output, they are also considered to provide the same information processing. Then, they are referred to as transformation-equivalent (T-equivalent). It has been reported that a right QVMLP can be implemented using a left QVMLP [16]. This is the first example of T-equivalent MLPs. Then, it was theoretically clarified that the left and right QVMLPs had the same computational power, which had been reported only by computer simulations, and had never been revealed for a generation [17]. In addition, a few examples of transformations were provided for quaternion-valued Hopfield networks in [16]. Thus, it was found that T-equivalence was useful for understanding the mechanisms of learning machines. In this paper, we provide constructive families and procedures for obtaining T-equivalent networks of RVMLPs, CVMLPs and QVMLPs. T-equivalence of RVMLPs is really simple. In CVMLPs, the conjugate operator emerges, unlike in RVMLPs. In QVMLPs, we have to discuss the permutations of axes in 4D space. In particular, we will find that the T-equivalence of QVMLPs is complicated.

The rest of this paper is organized as follows. In Section 2, the MLPs are defined. I/O-equivalence is described in Section 3. Section 4 provides the definition of T-equivalence and discusses the T-equivalence of RVMLPs and CVMLPs. The T-equivalence of QVMLPs is complicated, since the left and right QVMLPs are different. We prepare the theory for QVMLPs in Section 5 and provide the procedures for obtaining T-equivalent QVMLPs in Section 6. Finally, Section 7 concludes this paper.

## 2. Multilayer Perceptrons

### 2.1. Real- and Complex-Valued Multilayer Perceptrons

We briefly describe RVMLPs and CVMLPs. We assume that the MLPs satisfy the following conditions:

- The MLPs are three-layer perceptrons with one output.
- All the neurons have no bias terms.
- The split activation functions are employed; the RVMLPs employ f(t)=tanh(t), and the CVMLPs employ f(s+ti)=tanh(s)+tanh(t)i.

For a vector $\vec{t}={\left(t_{1},\cdots ,t_{l}\right)}^{T}$, we denote $f\left(\vec{t}\right)={\left(f\left(t_{1}\right),\cdots ,f\left(t_{l}\right)\right)}^{T}$ and $f\left({\vec{t}}^{\;T}\right)=\left(f\left(t_{1}\right),\cdots ,f\left(t_{l}\right)\right)$. We define some symbols.

- *m* is the dimension of the input vectors.
- *n* is the number of hidden neurons.
- $\vec{x}={\left(x_{1},\cdots ,x_{m}\right)}^{T}$ is the input vector.
- ${\vec{u}}_{j}={\left(u_{j1},\cdots ,u_{jm}\right)}^{T}$ is the weight vector from the input layer to hidden neuron *j*; $u_{jk}$ is the weight from the *k*th element of the input vector. Moreover, we define the weight matrix $U={\left({\vec{u}}_{1}\;\cdots \;{\vec{u}}_{n}\right)}^{T}$ from the input layer to the output layer.
- $\vec{S}={\left(S_{1},\cdots ,S_{n}\right)}^{T}=U\vec{x}$ is the vector of weighted sum inputs to the hidden layer; $S_{j}={\vec{u}}_{j}\cdot \vec{x}$ is the weighted sum input to hidden neuron *j*. The dot means the inner product of vectors.
- $\vec{y}={\left(y_{1},\cdots ,y_{n}\right)}^{T}=f\left(\vec{S}\right)={\left(f\left(S_{1}\right),\cdots ,f\left(S_{n}\right)\right)}^{T}$ is the output vector of the hidden layer; $y_{j}=f\left(S_{j}\right)$ is the output of hidden neuron *j*.
- $\vec{v}={\left(v_{1},\cdots ,v_{n}\right)}^{T}$ is the weight vector from the hidden layer to the output neuron; $v_{j}$ is the weight from hidden neuron *j*.
- $z=f(\vec{v}\cdot \vec{y})$ is the output of the MLP. We consider that an MLP is an *m* variable function, and denote it as $z=F\left(\vec{x};U,\vec{v}\right)$ or simply $z=F\left(\vec{x}\right)$.

We denote the conjugate of complex number *t* as $t^{†}$. If *t* is a real number, $t^{†}=t$ holds. For a vector $\vec{t}=\left(t_{1},\cdots ,t_{l}\right)$, we denote ${\vec{t}}^{\;†}=\left(t_{1}^{†},\cdots ,t_{l}^{†}\right)$. For a matrix *A*, $A^{†}$ is defined in a similar way. The following equalities are obtained by direct calculations:

(1) $$\begin{array}{ccc} f(\rho t) & = & \rho f(t), \end{array}$$

(2) $$\begin{array}{ccc} f(t^{†}) & = & f{\left(t\right)}^{†}, \end{array}$$

where ρ is ±1 in RVMLPs and ±1 or ±i in CVMLPs. We can regard an MLP as a multivariable function; that is, $z=f(\vec{v}\cdot f\left(U\vec{x}\right))$. Since an MLP is determined by the weight parameters, it is also denoted as $F(\vec{x}\;;\;U,\vec{v})$, often $F(\vec{x})$ for short.

### 2.2. Quaternion-Valued Multilayer Perceptrons

A quaternion is represented as $q=q_{0}+q_{1}\;i+q_{2}\;j+q_{3}\;k$ with four real numbers $q_{0}$, $q_{1}$, $q_{2}$, and $q_{3}$. Three imaginary units *i*, *j*, and *k* satisfy the following properties:

1. $i^{2}=j^{2}=k^{2}=-1$.
2. ij=−ji=k, jk=−kj=i, and ki=−ik=j.

The set of quaternions is denoted as H. Moreover, we define the sets Λ={i,j,k} and $\Lambda^{+}=\left\{1,i,j,k\right\}$. The set $\pm \Lambda^{+}$ forms a group. Quaternions form a non-commutative field. The conjugate is extended to quaternions as $q^{†}=q_{0}-q_{1}\;i-q_{2}\;j-q_{3}\;k$. For the quaternion conjugate, the equality ${\left(qq^{\prime }\right)}^{†}=q^{\prime †}q^{†}$ holds. We provide some properties for matrices *A* and *B*. We denote the Hermitian transpose of *A*, that is, the conjugate and transpose, as $A^{H}$. The equality ${\left(AB\right)}^{H}=B^{H}A^{H}$ is true for both complex and quaternion matrices. On the other hand, the equalities ${\left(AB\right)}^{T}=B^{T}A^{T}$ and ${\left(AB\right)}^{†}=A^{†}B^{†}$ are true for complex matrices, but they are false for quaternion matrices. Let $\vec{a}$ and $\vec{b}$ be quaternion vectors. Then, the equality ${\left(\vec{a}\cdot \vec{b}\right)}^{†}={\vec{b}}^{\;†}\cdot {\vec{a}}^{\;†}$ holds.

We modify the CVMLPs to QVMLPs. The activation function is defined as

(3) $$\begin{array}{ccc} f(q_{0}+q_{1}\;i+q_{2}\;j+q_{3}\;k) & = & \tanh\left(q_{0}\right)+\tanh\left(q_{1}\right)\;i+\tanh\left(q_{2}\right)\;j+\tanh\left(q_{3}\right)\;k. \end{array}$$

The equalities (1) and (2) hold for the quaternion-valued activation function. Most of definitions in Section 2.1 are also employed for QVMLPs. However, the definition of weighed sum inputs is necessarily modified, since quaternions are not commutative. QVMLPs are classified as left and right QVMLPs. The weighted sum inputs ${\vec{u}}_{j}\cdot \vec{x}$ and $\vec{x}\cdot {\vec{u}}_{j}$ are different, unlike with RVMLPs and CVMLPs. The left and right QVMLPs employ $S_{j}={\vec{u}}_{j}\cdot \vec{x}$ and $S_{j}=\vec{x}\cdot {\vec{u}}_{j}$, respectively. Therefore, $\vec{S}$ is represented as ${\vec{S}}^{\;T}={\vec{x}}^{\;T}U^{T}$ for the right QVMLPs. We should note $\vec{S}={\left({\vec{x}}^{\;T}U^{T}\right)}^{T}\neq U\vec{x}$. Similarly, the weighted sum input to the output neuron is modified to $\vec{y}\cdot \vec{v}$ for right QVMLPs. Left and right QVMLPs are denoted as $z=F_{L}\left(\vec{x}\right)$ and $z=F_{R}\left(\vec{x}\right)$, respectively;

(4) $$\begin{array}{ccc} F_{L}\left(\vec{x};U,\vec{v}\;\right) & = & f\left(\vec{v}\cdot f\left(U\vec{x}\;\right)\right)\;\;=\;\;f\left({\vec{v}}^{T}f\left(U\vec{x}\;\right)\right), \end{array}$$

(5) $$\begin{array}{ccc} F_{R}\left(\vec{x};U,\vec{v}\;\right) & = & f\left(f\left({\left({\vec{x}}^{\;T}U^{T}\right)}^{T}\right)\cdot \vec{v}\;\right)\;\;=\;\;f\left(f\left({\vec{x}}^{\;T}U^{T}\right)\vec{v}\;\right). \end{array}$$

## 3. I/O-Equivalence of Multilayer Perceptrons

**Figure 1:**
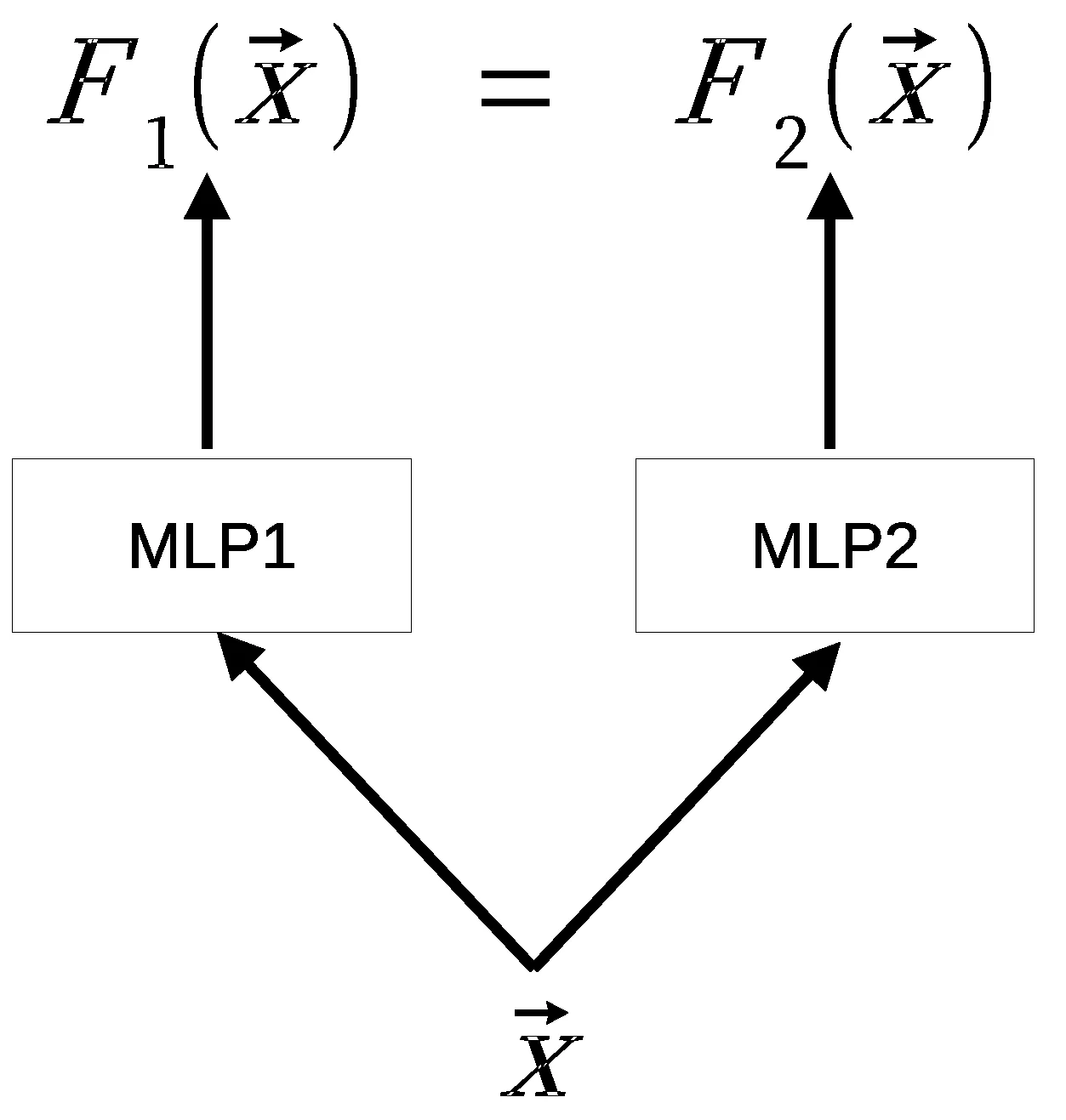
This figure illustrates I/O-equivalence. Two MLPs are referred to as I/O-equivalent if their I/Os are identical.

We provide an overview of the I/O-equivalence of MLPs. We consider two MLPs and denote them as $F_{1}\left(\vec{x}\right)$ and $F_{2}\left(\vec{x}\right)$. If they satisfy $F_{1}\left(\vec{x}\right)=F_{2}\left(\vec{x}\right)$ for all $\vec{x}$, they are referred to as I/O-equivalent. Figure 1 illustrates I/O-equivalence. I/O-equivalence is clearly an equivalent relation. For two I/O-equivalent MLPs, the numbers of hidden neurons may be different. For example, we consider an MLP with $v_{n}=0$. Then, even if hidden neuron *n* is removed, the I/O of the MLP maintains. The MLP $F(\vec{x})$ is referred to as minimal if there exist no MLPs with fewer hidden neurons, such that they are I/O-equivalent to $F(\vec{x})$ itself. An RVMLP is transformed to an I/O-equivalent minimal with three types of procedures [1]. If there are no bias terms, CVMLPs are transformed to be minimal using similar methods [8]. If CVMLPs have bias terms, there are exceptional cases [10]. QVMLPs with no bias terms can also be transformed to be minimal [15]. Hereafter, we consider only minimal MLPs; *n* is fixed. The conditions such that two MLPs are I/O-equivalent have already been determined [1,8,10]. We explain two procedures to obtain I/O-equivalent MLPs in RVMLPs and CVMLPs.

(1) To permute the hidden neurons, we define the matrix $P=\left(\begin{array}{ccc} \delta_{1\sigma (1)} & \cdots & \delta_{1\sigma (n)}\\ \vdots & \ddots & \vdots \\ \delta_{n\sigma (1)} & \cdots & \delta_{n\sigma (n)} \end{array}\right)$ with a permutation σ, where $\delta_{ab}$ is the Kronecker delta. The transformation is represented by $\left(U,\vec{v}\;\right)\mapsto \left(PU,P\vec{v}\;\right)$. We should note that *P* is an orthogonal matrix.
(2) To transform *U* and $\vec{v}$ using $\rho_{a}\;\left(a=1,2,\cdots ,n\right)$, where $\rho_{a}$ is ±1 for RVMLPs, and ±1 or ±i for CVMLPs, we define the matrix $D=\left(\begin{array}{ccc} \rho_{1} & \cdots & 0\\ \vdots & \ddots & \vdots \\ 0 & \cdots & \rho_{n} \end{array}\right).$ The transformation is represented by $\left(U,\vec{v}\;\right)\mapsto \left(DU,D^{†}\vec{v}\;\right)$.

We can obtain I/O-equivalent MLPs by these procedures; $F(\vec{x}\;;\;U,\vec{v}\;)$ and $F(\vec{x}\;;\;DPU,D^{†}P\vec{v}\;)$ are I/O-equivalent.

Procedure (1) is available for QVMLPs, while we have to modify procedure (2) [15]. In both the left and right QVMLPs, $\rho_{a}$ belongs to ±Λ. For the left QVMLPs, the procedure (2) is modified to $\left(U,\vec{v}\;\right)\mapsto \left(DU,{\left({\vec{v}}^{T}\;D^{†}\right)}^{T}\right)$; QVMLPs $F_{L}\left(\vec{x}\;;\;U,\vec{v}\;\right)$ and $F_{L}\left(\vec{x}\;;\;DPU,{\left({\left(P\vec{v}\;\right)}^{T}D^{†}\right)}^{T}\right)$ are I/O-equivalent. We note ${\left(P\vec{v}\;\right)}^{T}={\vec{v}}^{\;T}P^{T}$, since *P* is a real matrix. We obtain

(6) $$\begin{array}{ccc} f\left({\left(P\vec{v}\;\right)}^{T}D^{†}f\left(DPU\vec{x}\;\right)\right) & = & f\left({\left(P\vec{v}\;\right)}^{T}D^{†}Df\left(PU\vec{x}\;\right)\right) \end{array}$$

(7) $$\begin{array}{ccc} & = & f\left({\vec{v}}^{\;T}P^{T}Pf\left(U\vec{x}\;\right)\right) \end{array}$$

(8) $$\begin{array}{ccc} & = & f\left({\vec{v}}^{\;T}f\left(U\vec{x}\;\right)\right). \end{array}$$

For right QVMLPs, it is modified to $\left(U,\vec{v}\;\right)\mapsto \left({\left(U^{T}D\right)}^{T},D^{†}\vec{v}\;\right)$; QVMLPs $F_{R}\left(\vec{x}\;;\;U,\vec{v}\;\right)$ and $F_{R}\left(\vec{x}\;;\;{\left({\left(PU\right)}^{T}D\right)}^{T},D^{†}P\vec{v}\right)$ are I/O-equivalent. In fact, we obtain

(9) $$\begin{array}{ccc} f\left(f\left({\vec{x}}^{\;T}{\left(PU\right)}^{T}D\right)\left(D^{†}P\vec{v}\;\right)\right) & = & f\left(f\left({\vec{x}}^{T}U^{T}P^{T}\right)\left(P\vec{v}\;\right)\right) \end{array}$$

(10) $$\begin{array}{ccc} & = & f\left(f\left({\vec{x}}^{T}U^{T}\right)\vec{v}\;\right). \end{array}$$

## 4. Transformation Equivalence of Real- and Complex-Valued MLPs

**Figure 2:**
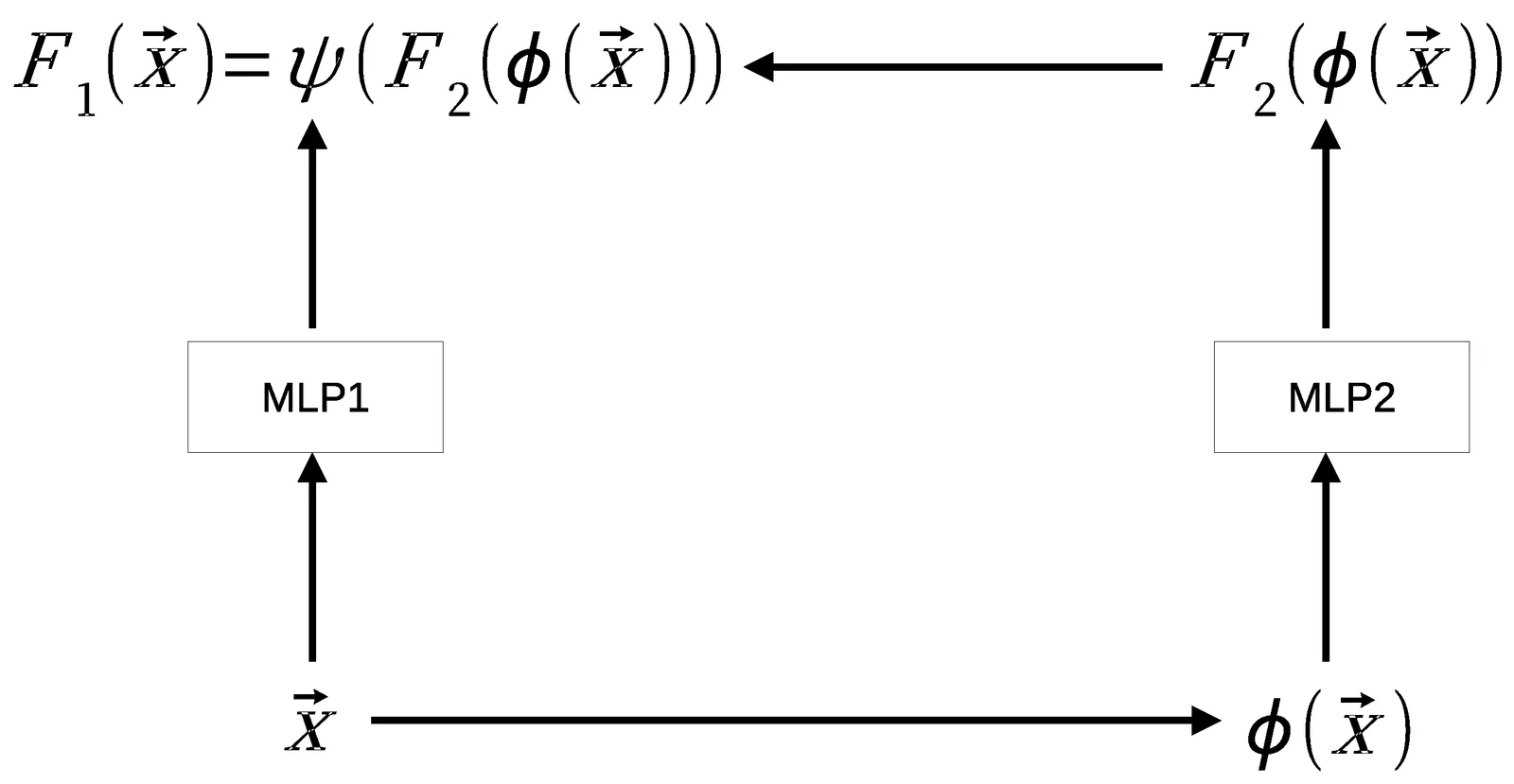
This figure illustrates T-equivalence. Two MLPs are referred to as T-equivalent if their I/Os are identical through transformation.

We consider a problem where we get the output of $F\left(\vec{x}\right)$ using another MLP which is not I/O-equivalent to $F\left(\vec{x}\right)$. It has been reported that a QVMLP can be implemented using the other type of QVMLP [16]. For two MLPs $F_{1}\left(\vec{x}\right)$ and $F_{2}\left(\vec{x}\right)$, if there exist invertible maps $\phi (\vec{x})$ and ψ(z) such that $F_{1}\left(\vec{x}\right)=\psi \left(F_{2}\left(\phi \left(\vec{x}\right)\right)\right)$, $F_{1}\left(\vec{x}\right)$ is T-equivalent to $F_{2}\left(\vec{x}\right)$ with $\phi (\vec{x})$ and ψ(z). Then, $F_{2}\left(\vec{x}\right)$ is also T-equivalent to $F_{1}\left(\vec{x}\right)$ with $\phi^{-1}\left(\vec{x}\right)$ and $\psi^{-1}\left(z\right)$. If $F_{1}\left(\vec{x}\right)$ is T-equivalent to $F_{2}\left(\vec{x}\right)$ with $\phi_{1}\left(\vec{x}\right)$ and $\psi_{1}\left(z\right)$ and $F_{2}\left(\vec{x}\right)$ is T-equivalent to $F_{3}\left(\vec{x}\right)$ with $\phi_{2}\left(\vec{x}\right)$ and $\psi_{2}\left(z\right)$, then $F_{1}\left(\vec{x}\right)$ is T-equivalent to $F_{3}\left(\vec{x}\right)$ with $\phi_{2}\left(\phi_{1}\left(\vec{x}\right)\right)$ and $\psi_{1}\left(\psi_{2}\left(z\right)\right)$. In particular, I/O-equivalence is T-equivalence with $\phi \left(\vec{x}\;\right)=\vec{x}$ and ψ(z)=z. We add some procedures to extend I/O-equivalence to T-equivalence. Figure 2 illustrates I/O-equivalence.

We consider the T-equivalence of RVMLPs. We take $\rho_{1}=\pm 1$, $\rho_{2}=\pm 1$, and $A\in {\mathrm{GL}}_{m}\left(R\right)$.

(11) $$\begin{array}{ccc} F\left(A\vec{x};\rho_{1}UA^{-1},\rho_{2}\vec{v}\right) & = & f\left(\left(\rho_{2}\vec{v}\right)\cdot f\left(\rho_{1}UA^{-1}A\vec{x}\right)\right) \end{array}$$

(12) $$\begin{array}{ccc} & = & \rho_{1}\rho_{2}f\left(\vec{v}\cdot f\left(U\vec{x}\right)\right) \end{array}$$

(13) $$\begin{array}{ccc} & = & \rho_{1}\rho_{2}\;F\left(\vec{x};U,\vec{v}\right) \end{array}$$

Therefore, $F\left(\vec{x};U,\vec{v}\right)$ is T-equivalent to $F\left(\vec{x};\rho_{1}UA^{-1},\rho_{2}\vec{v}\right)$ with $\phi \left(\vec{x}\right)=A\vec{x}$ and $\psi \left(z\right)=\rho_{1}\rho_{2}z$.

**Figure 3:**
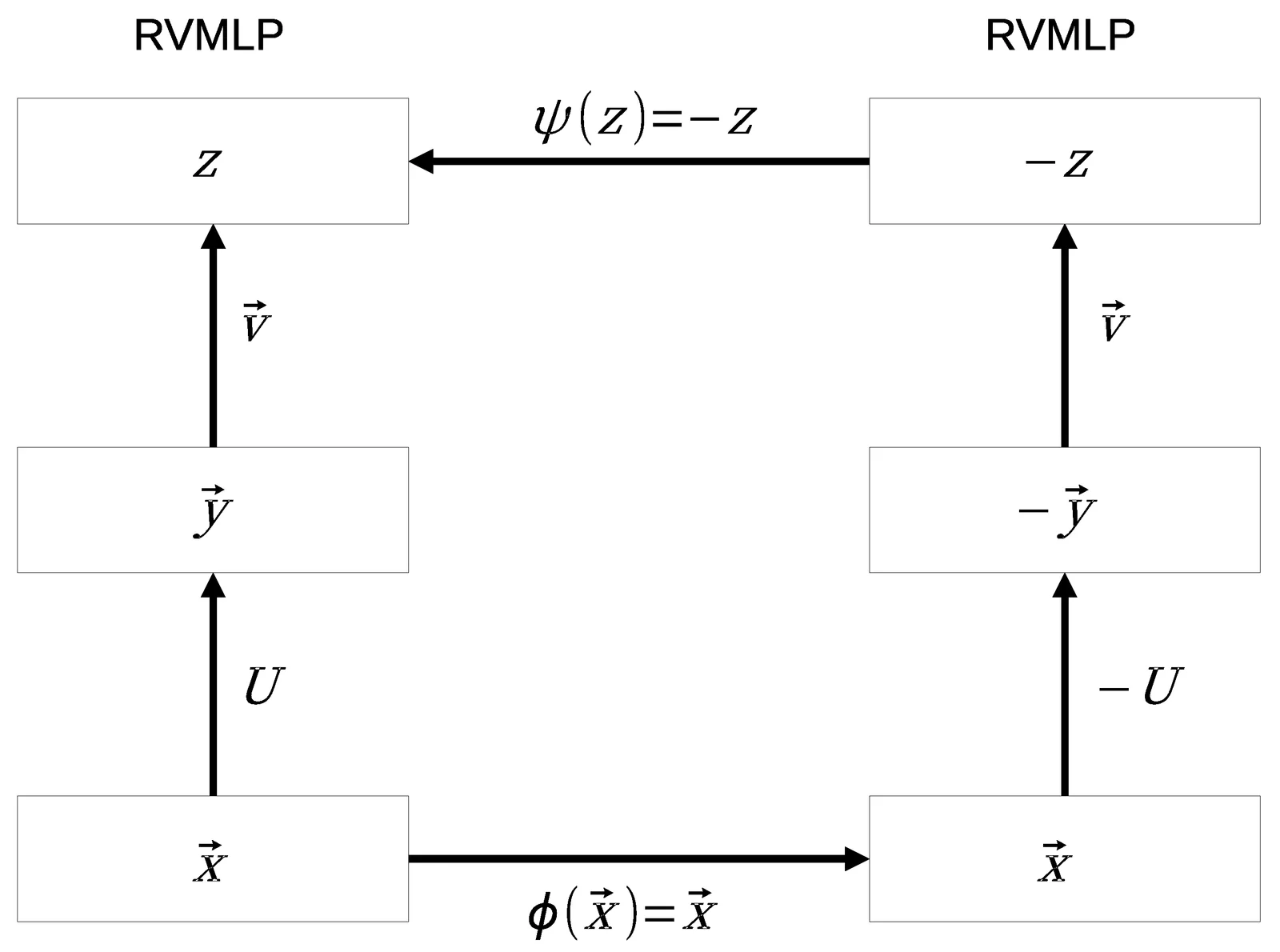
$F\left(\vec{x};U,\vec{v}\right)$ is T-equivalent to $F\left(\vec{x};-U,\vec{v}\right)$ with $\phi \left(\vec{x}\right)=\vec{x}$ and ψ(z)=−z.

**Example** **1.** *$F\left(\vec{x};U,\vec{v}\right)$ is T-equivalent to $F\left(\vec{x};-U,\vec{v}\right)$ with $\phi \left(\vec{x}\right)=\vec{x}$ and ψ(z)=−z (Figure 3).*

We extend the T-equivalence of RVMLPs to that of CVMLPs. We take $\rho_{1},\rho_{2}\in \left\{\pm 1,\pm i\right\}$, and $A\in {\mathrm{GL}}_{m}\left(C\right)$. We can show $F\left(A\vec{x};\rho_{1}UA^{-1},\rho_{2}\vec{v}\right)=\rho_{1}\rho_{2}F\left(\vec{x};U,\vec{v}\right)$ in the same way as RVMLPs. Therefore, $F\left(\vec{x};U,\vec{v}\right)$ is T-equivalent to $F\left(\vec{x};\rho_{1}UA^{-1},\rho_{2}\vec{v}\right)$ with $\phi \left(\vec{x}\right)=A\vec{x}$ and $\psi \left(z\right)={\left(\rho_{1}\rho_{2}\right)}^{†}z$. We provide a different type of T-equivalence.

(14) $$\begin{array}{ccc} F\left(A{\vec{x}}^{\;†};\rho_{1}U^{†}A^{-1},\rho_{2}{\vec{v}}^{\;†}\right) & = & f\left(\left(\rho_{2}{\vec{v}}^{\;†}\right)\cdot f\left(\rho_{1}U^{†}A^{-1}A{\vec{x}}^{\;†}\right)\right) \end{array}$$

(15) $$\begin{array}{ccc} & = & \rho_{1}\rho_{2}f{\left(\vec{v}\cdot f\left(U\vec{x}\right)\right)}^{†} \end{array}$$

(16) $$\begin{array}{ccc} & = & \rho_{1}\rho_{2}\;F{\left(\vec{x};U,\vec{v}\right)}^{†} \end{array}$$

Thus, $F\left(\vec{x};U,\vec{v}\right)$ is T-equivalent to $F\left(\vec{x};\rho_{1}U^{†}A^{-1},\rho_{2}{\vec{v}}^{†}\right)$ with $\phi \left(\vec{x}\right)=A{\vec{x}}^{\;†}$ and $\psi \left(z\right)=\rho_{1}\rho_{2}z^{†}$.

**Figure 4:**
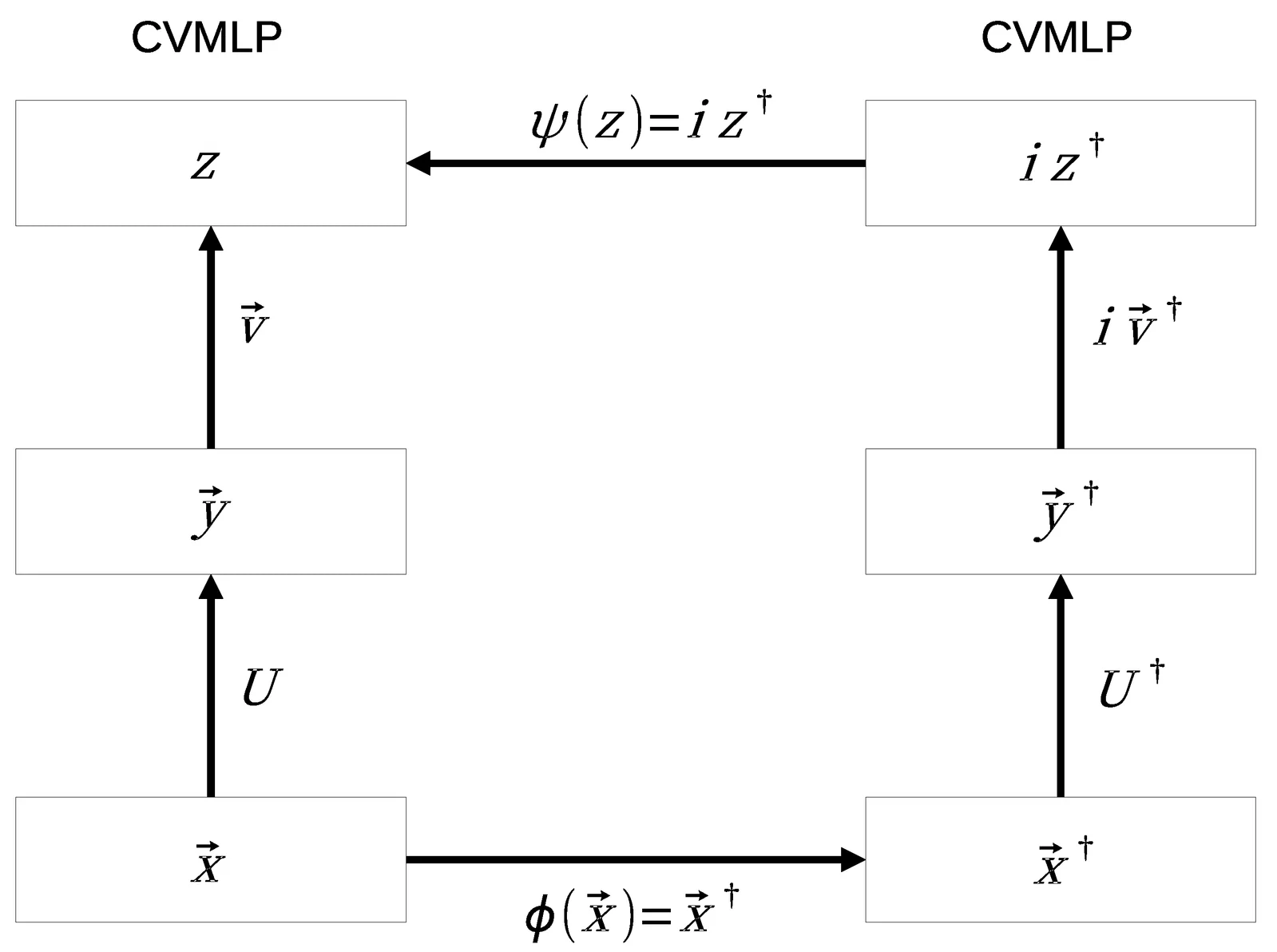
$F\left(\vec{x};U,\vec{v}\right)$ is T-equivalent to $F\left(\vec{x};U^{\;†},i{\vec{v}}^{\;†}\right)$ with $\phi \left(\vec{x}\right)={\vec{x}}^{\;†}$ and $\psi \left(z\right)=iz^{†}$.

**Example** **2.** *$F\left(\vec{x};U,\vec{v}\right)$ is T-equivalent to $F\left(\vec{x};U^{\;†},i{\vec{v}}^{\;†}\right)$ with $\phi \left(\vec{x}\right)={\vec{x}}^{\;†}$ and $\psi \left(z\right)=iz^{†}$ (Figure 4).*

## 5. Operators on Quaternions

We discuss operators on quaternions. We consider the quaternion $q=q_{0}+q_{1}\;i+q_{2}\;j+q_{3}\;k$ as the 4D vector $\vec{q}={\left(q_{0},q_{1},q_{2},q_{3}\right)}^{T}$. For $\alpha =\alpha_{0}+\alpha_{1}\;i+\alpha_{2}\;j+\alpha_{3}\;k\neq 0$, the action αq from the left is represented by $Q_{L}\left(\alpha \right)\;\vec{q}$ with

(17) $$\begin{array}{ccc} Q_{L}\left(\alpha \right) & = & \left(\begin{array}{cccc} \alpha_{0} & -\alpha_{1} & -\alpha_{2} & -\alpha_{3}\\ \alpha_{1} & \alpha_{0} & -\alpha_{3} & \alpha_{2}\\ \alpha_{2} & \alpha_{3} & \alpha_{0} & -\alpha_{1}\\ \alpha_{3} & -\alpha_{2} & \alpha_{1} & \alpha_{0} \end{array}\right), \end{array}$$

and the action qα from the right is represented by $Q_{R}\left(\alpha \right)\vec{q}$ with

(18) $$\begin{array}{ccc} Q_{R}\left(\alpha \right) & = & \left(\begin{array}{cccc} \alpha_{0} & -\alpha_{1} & -\alpha_{2} & -\alpha_{3}\\ \alpha_{1} & \alpha_{0} & \alpha_{3} & -\alpha_{2}\\ \alpha_{2} & -\alpha_{3} & \alpha_{0} & \alpha_{1}\\ \alpha_{3} & \alpha_{2} & -\alpha_{1} & \alpha_{0} \end{array}\right). \end{array}$$

The determinants of $Q_{L}\left(\alpha \right)$ and $Q_{R}\left(\alpha \right)$ are $\left|\alpha \right|=\sqrt{\alpha_{0}^{2}+\alpha_{1}^{2}+\alpha_{2}^{2}+\alpha_{3}^{2}}$. In particular, they are positive. Therefore, $Q_{L}\left(\alpha \right)$ and $Q_{R}\left(\alpha \right)$ keep the direction of the coordinate system. We provide some properties. The following lemma provides the properties of $Q_{L}$ and $Q_{R}$.

**Lemma** **1.**
(19) $$\begin{array}{ccc} Q_{L}\left(\alpha \right)\;Q_{R}\left(\beta \right) & = & Q_{R}\left(\beta \right)\;Q_{L}\left(\alpha \right) \end{array}$$

(20) $$\begin{array}{ccc} Q_{L}\left(\alpha \beta \right) & = & Q_{L}\left(\alpha \right)\;Q_{L}\left(\beta \right) \end{array}$$

(21) $$\begin{array}{ccc} Q_{R}\left(\alpha \beta \right) & = & Q_{R}\left(\beta \right)\;Q_{R}\left(\alpha \right) \end{array}$$

**Proof.**  The first equality corresponds to the associative law α(qβ)=(αq)β. The second and third equalities follow the associative laws (αβ)q=α(βq) and q(αβ)=(qα)β again. □

The conjugate operation $q^{†}$ is represented by $C\;\vec{q}$ with

(22) $$\begin{array}{ccc} C & = & \left(\begin{array}{cccc} 1 & 0 & 0 & 0\\ 0 & -1 & 0 & 0\\ 0 & 0 & -1 & 0\\ 0 & 0 & 0 & -1 \end{array}\right), \end{array}$$

and *C* changes the direction of the coordinate system because of det(C)=−1. In particular, −C changes only the direction of the real axis. The following lemma provides the properties of $Q_{L}$, $Q_{R}$ and *C*.

**Lemma** **2.**
(23) $$\begin{array}{ccc} C^{2} & = & I \end{array}$$

(24) $$\begin{array}{ccc} C\;Q_{L}\left(\alpha \right) & = & Q_{R}\left(\alpha^{†}\right)\;C \end{array}$$

(25) $$\begin{array}{ccc} C\;Q_{R}\left(\alpha \right) & = & Q_{L}\left(\alpha^{†}\right)\;C \end{array}$$

**Proof.**  The first equality corresponds to $q^{††}=q$. The second and third equalities follow ${\left(\alpha q\right)}^{†}=q^{†}\alpha^{†}$ and ${\left(q\alpha \right)}^{†}=\alpha^{†}q^{†}$. □

Three imaginary axes generate the 3D space, referred to as imaginary space. We describe several rotation transformations in the imaginary space, fixing the real axis [18]. We denote the rotation around the *l*-axis anticlockwise at an angle θ as $R_{l}\left(\theta \right)$ with l∈Λ. The rotations $R_{l}\left(\pi \right)$ and $R_{l}\left(\frac{\pi }{2}\right)$ are induced by the operations lq(−l) and $\left(\frac{1+l}{\sqrt{2}}\right)q\left(\frac{1-l}{\sqrt{2}}\right)$, respectively:

(26) $$\begin{array}{ccc} R_{l}\left(\pi \right) & = & Q_{L}\left(l\right)\;Q_{R}\left(-l\right), \end{array}$$

(27) $$\begin{array}{ccc} R_{l}\left(\frac{\pi }{2}\right) & = & Q_{L}\left(\frac{1+l}{\sqrt{2}}\right)Q_{R}\left(\frac{1-l}{\sqrt{2}}\right). \end{array}$$

Then, the following equalities hold:

(28) $$\begin{array}{ccc} R_{l}\left(\pi \right) & = & R_{l}\left(-\pi \right)\;=\;R_{l}{\left(\frac{\pi }{2}\right)}^{2}, \end{array}$$

(29) $$\begin{array}{ccc} R_{l}\left(\frac{3\pi }{2}\right) & = & R_{l}\left(-\frac{\pi }{2}\right)\;=\;R_{l}{\left(\frac{\pi }{2}\right)}^{3}. \end{array}$$

We extend the notation to $R_{-l}\left(\theta \right)=R_{l}\left(-\theta \right)$; then, (27) is also extended to

(30) $$\begin{array}{ccc} R_{\pm l}\left(\frac{\pi }{2}\right) & = & Q_{L}\left(\frac{1\pm l}{\sqrt{2}}\right)Q_{R}\left(\frac{1\mp l}{\sqrt{2}}\right). \end{array}$$

**Example** **3.** *We consider the case of l=i.*

(31) $$\begin{array}{ccc} iq(-i) & = & q_{0}+q_{1}i-q_{2}j-q_{3}k \end{array}$$

(32) $$\begin{array}{ccc} R_{i}\left(\pi \right) & = & Q_{L}\left(i\right)\;Q_{R}\left(-i\right) \end{array}$$

(33) $$\begin{array}{ccc} & = & \left(\begin{array}{cccc} 1 & 0 & 0 & 0\\ 0 & 1 & 0 & 0\\ 0 & 0 & -1 & 0\\ 0 & 0 & 0 & -1 \end{array}\right) \end{array}$$

(34) $$\begin{array}{ccc} \left(\frac{1+i}{\sqrt{2}}\right)q\left(\frac{1-i}{\sqrt{2}}\right) & = & q_{0}+q_{1}i-q_{3}j+q_{2}k \end{array}$$

(35) $$\begin{array}{ccc} R_{i}\left(\frac{\pi }{2}\right) & = & Q_{L}\left(\frac{1+i}{\sqrt{2}}\right)Q_{R}\left(\frac{1-i}{\sqrt{2}}\right) \end{array}$$

(36) $$\begin{array}{ccc} & = & \left(\begin{array}{cccc} 1 & 0 & 0 & 0\\ 0 & 1 & 0 & 0\\ 0 & 0 & 0 & -1\\ 0 & 0 & 1 & 0 \end{array}\right) \end{array}$$

We determine the permutations of axes by the following steps, where the direction changes of axes are included.

(A) We determine the permutations of imaginary axes; the real axis is fixed. After we determine the permutations keeping the direction of the coordinate system, we determine all the permutations.
(B) After the real and one of the imaginary axes are exchanged, step (A) is conducted.
(C) After step (B), the real axis is reversed.

**Figure 5:**
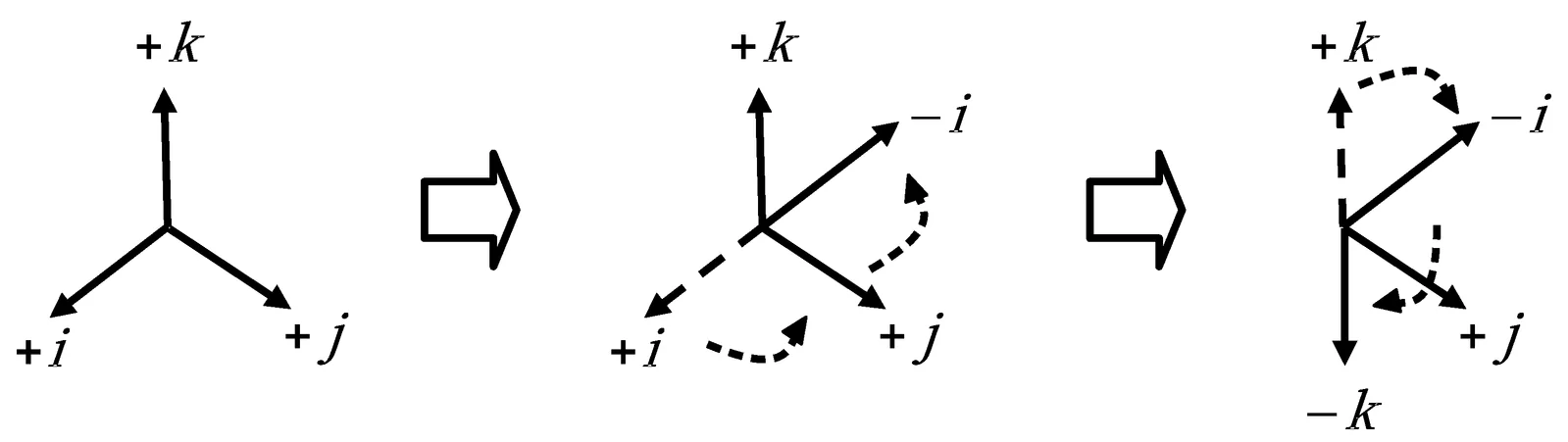
This figure illustrates the first process of step (A). One of the imaginary axes is selected, and the imaginary space is rotated around the selected axis by $\frac{\pi }{2}$. All the permutations of imaginary axes keeping the direction of the coordinate system are obtained by repeating this operation. In this figure, the first operation is the anticlockwise rotation around *k*-axis, and the second one is the clockwise rotation around *j*-axis.

**Figure 6:**
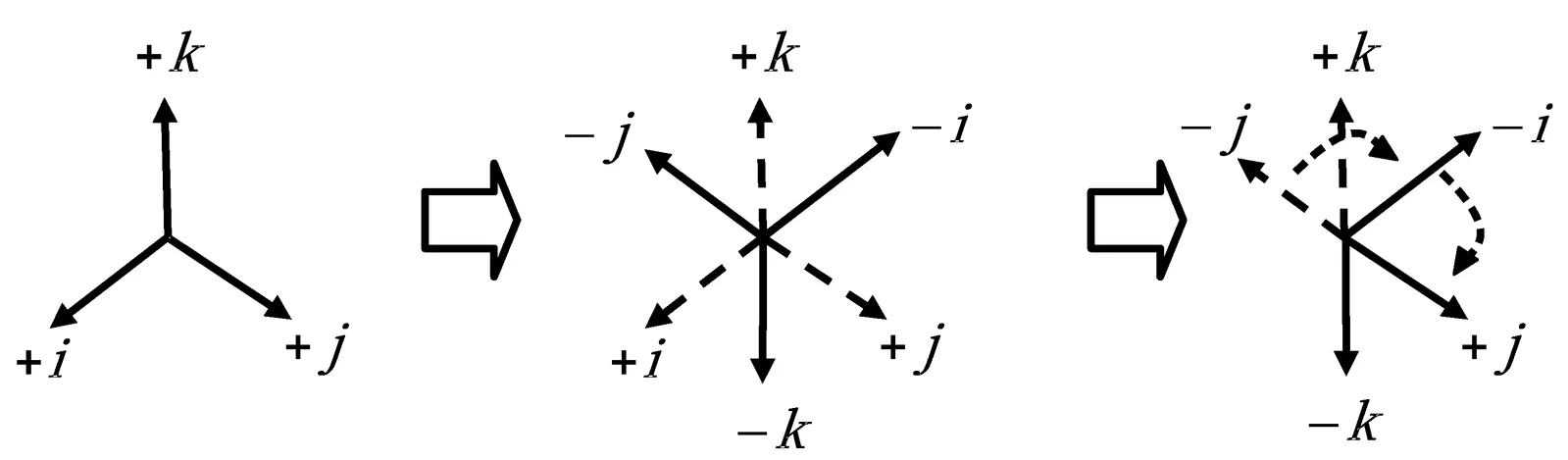
This figure illustrates the second process of step (A). The left operation shows that all signs of imaginary components are reversed by conjugate operator *C*. This operation changes the direction of the coordinate system. After that, the first process is conducted. In this figure, the second operation is the anticlockwise rotation around the *k*-axis.

**Figure 7:**
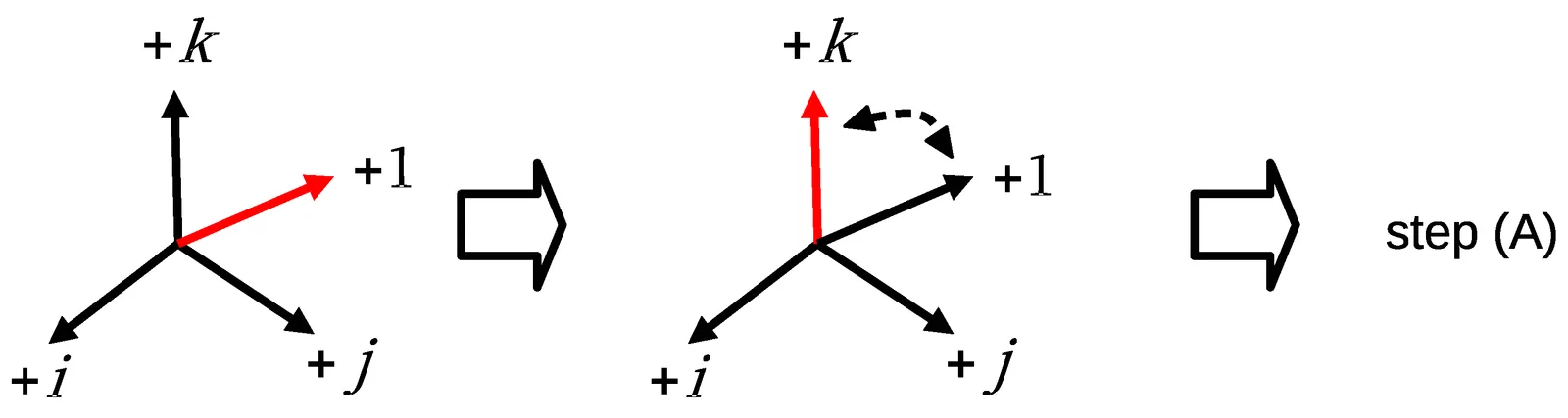
This figure illustrates step (B). One of the imaginary axes and the real axis are exchanged. This figure shows the exchange of *k* and the real axes. After that, step (A) is conducted.

**Figure 8:**
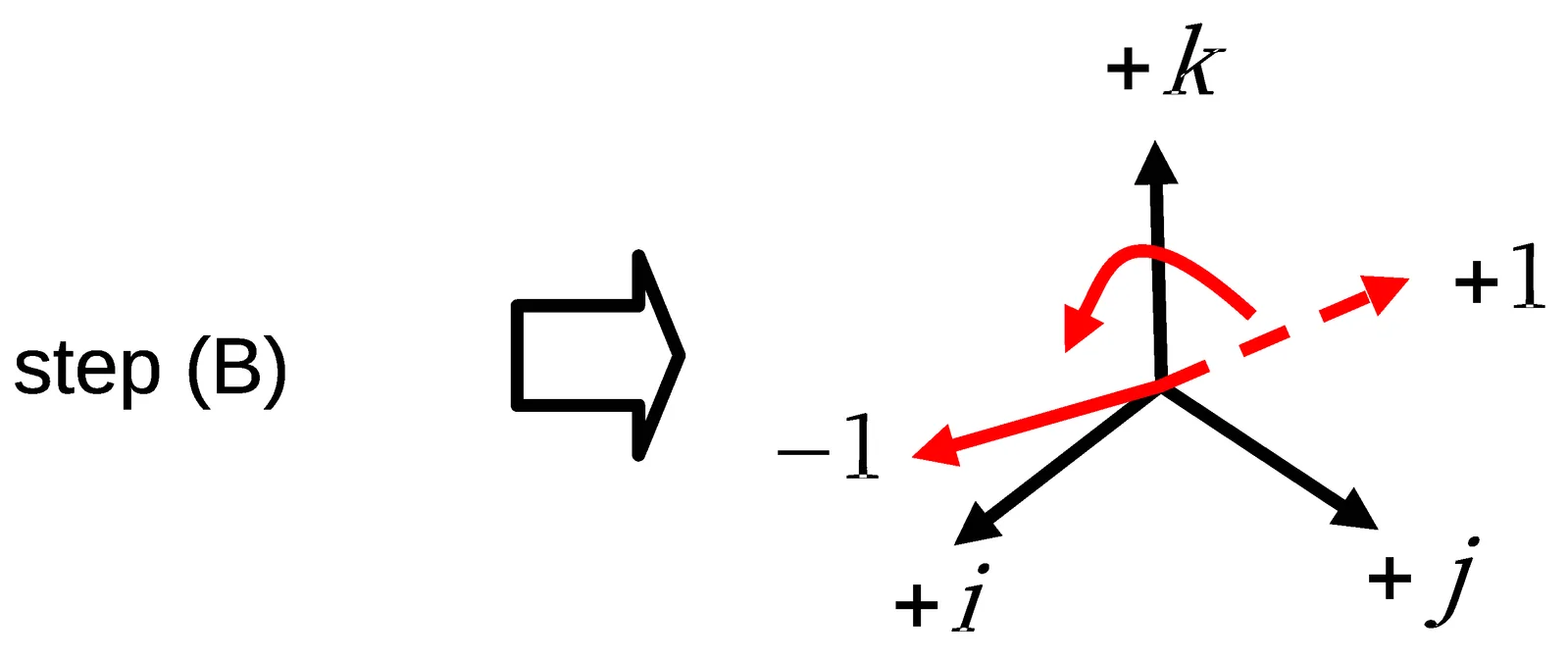
This figure illustrates step (C). After step (B), the sign of the real component is reversed by operator −C. This operation changes the direction of the coordinate system.

Figure 5, Figure 6, Figure 7 and Figure 8 illustrate these steps. Step (A) consists of two processes.

First, we determine the operators of step (A). We consider the rotations of imaginary axes, keeping the direction of the coordinate system. We note that, when the destinations of the *i*- and *j*-axes are determined, the destination of the *k*-axis is also determined, since the direction of the coordinate system is kept. The numbers of destinations of the *i*- and *j*-axes are 6 and 4, respectively; the number of rotations is 6×4=24. We consider the rotations generated by ${\left\{R_{l}\left(\frac{\pi }{2}\right)\right\}}_{l=i,j,k}$. We define the set

(37) $$\begin{array}{ccc} \Omega & = & \left\{1,i,j,k,\frac{1\pm i}{\sqrt{2}},\frac{1\pm j}{\sqrt{2}},\frac{1\pm k}{\sqrt{2}},\frac{i\pm j}{\sqrt{2}},\frac{j\pm k}{\sqrt{2}},\frac{k\pm i}{\sqrt{2}},\frac{1\pm i\pm j\pm k}{2}\right\}. \end{array}$$

Then, ±Ω is the group generated by ${\left\{\frac{1+l}{\sqrt{2}}\right\}}_{l=i,j,k}$ and is closed under conjugate. Let R(α) be the representation of operation $\alpha z\alpha^{†}$ for α∈±Ω. We determine step (A) using the following proposition.

**Proposition** **1.** *The permutations of imaginary axes are represented by $\left\{R(\alpha ),C\;R(\alpha )\;;\;\alpha \in \Omega \right\}$. Operator R(α) keeps the direction of the coordinate system, and operator CR(α) changes it.*

**Proof.**  We note R(α)=R(−α). From |Ω|=24, $\left\{R(\alpha );\alpha \in \Omega \right\}$ is the set of all the representations of rotations. Thus, we have determined all the permutations of imaginary axes, keeping the real axis and the direction of the coordinate system. Since the conjugate operation changes the direction of the coordinate system and fixes the real axis, $\left\{R(\alpha ),C\;R(\alpha );\alpha \in \Omega \right\}$ is the set of all the representations of permutations of the imaginary axes. □

We provide the relationship between R(α) and *C*.

**Lemma** **3.** *The operators R(α) and C are commutative:*

(38) $$\begin{array}{ccc} R(\alpha )\;C & = & C\;R(\alpha ). \end{array}$$

**Proof.**  From (24) and (25), we obtain

(39) $$\begin{array}{ccc} R(\alpha )\;C & = & Q_{L}\left(\alpha \right)\;Q_{R}\left(\alpha^{†}\right)\;C \end{array}$$

(40) $$\begin{array}{ccc} & = & C\;Q_{R}\left(\alpha^{†}\right)\;Q_{L}\left(\alpha \right) \end{array}$$

(41) $$\begin{array}{ccc} & = & C\;R(\alpha ). \end{array}$$

□

We add some properties about R(α).

**Lemma** **4.** *For α,β∈±Ω and $h\in \pm \Lambda^{+}$, the following equalities hold.*

(42) $$\begin{array}{ccc} R(\alpha \;\beta ) & = & R(\alpha )\;R(\beta ) \end{array}$$

(43) $$\begin{array}{ccc} R\left(\alpha \right)\;Q_{L}\left(h\right) & = & Q_{L}\left(\alpha h\alpha^{†}\right)R\left(\alpha \right) \end{array}$$

(44) $$\begin{array}{ccc} R\left(\alpha \right)\;Q_{R}\left(h\right) & = & Q_{R}\left(\alpha h\alpha^{†}\right)R\left(\alpha \right) \end{array}$$

*Moreover, $\alpha h\alpha^{†}$ belongs to ±Λ.*

**Proof.**  The first equality follows $\left(\alpha \beta \right)\;q\;{\left(\alpha \beta \right)}^{†}=\alpha \left(\beta \;q\;\beta^{†}\right)\alpha^{†}$. The second and third equalities are obtained from $\alpha hq\alpha^{†}=\left(\alpha h\alpha^{†}\right)\left(\alpha q\alpha^{†}\right)$ and $\alpha qh\alpha^{†}=\left(\alpha q\alpha^{†}\right)\left(\alpha h\alpha^{†}\right)$, respectively. Since operator R(α) permutes imaginary axes, $\alpha h\alpha^{†}$ belongs to ±Λ. □

**Lemma** **5.** *For α∈±Ω and $h\in \pm \Lambda^{+}$, the following equalities hold.*

(45) $$\begin{array}{ccc} Q_{R}\left(h\right)\;R\left(\alpha \right) & = & Q_{L}\left(h\right)\;R\left(h^{†}\alpha \right) \end{array}$$

(46) $$\begin{array}{ccc} Q_{L}\left(h\right)\;R\left(\alpha \right) & = & Q_{R}\left(h\right)\;R\left(h\alpha \right) \end{array}$$

*Moreover, $h^{†}\alpha$ and hα belong to ±Ω.*

**Proof.**  The first and second equalities are obtained from

(47) $$\begin{array}{ccc} (\alpha q\alpha^{†})h & = & h(\left(h^{†}\alpha \right)q{\left(h^{†}\alpha \right)}^{†}) \end{array}$$

and

(48) $$\begin{array}{ccc} h(\alpha q\alpha^{†}) & = & (\left(h\alpha \right)q{\left(h\alpha \right)}^{†})h, \end{array}$$

respectively. From $\pm \Lambda^{+}\subset \pm \Omega$, $h^{†}\alpha$ and hα belong to ±Ω. □

Next, we determine the operators of step (B).

**Proposition** **2.** *The operators of step (B) are represented as $Q_{L}\left(h\right)\;R\left(\alpha \right)$ or $C\;Q_{L}\left(h\right)\;R\left(\alpha \right)$ with h∈±Λ and α∈±Ω.*

We should note that all of $Q_{L}\left(h\right)\;R\left(\alpha \right)$ and $C\;Q_{L}\left(h\right)\;R\left(\alpha \right)$ do not represent step (B).

**Proof.**  The exchange of real and *l*-axes, that is, (1,l)↦(l,1), is represented by $R\left(\frac{1+l}{\sqrt{2}}\right)Q_{L}\left(l\right)\;C$. For example, we take l=i.

(49) $$\begin{array}{ccc} \left(\frac{1+i}{\sqrt{2}}\right)i\;\left(q_{0}-q_{1}\;i-q_{2}\;j-q_{3}\;k\right)\;\left(\frac{1-i}{\sqrt{2}}\right) & = & q_{1}+q_{0}\;i+q_{2}\;j+q_{3}\;k \end{array}$$

The transformation of step (A) is represented as R(α) or R(α)C with α∈Ω. We apply R(α) or R(α)C after the real and *l*-axes are exchanged. We put $\beta =\frac{\alpha (1+l)}{\sqrt{2}}\in \pm \Omega$. From Lemmas 2–5, we obtain the equalities

(50) $$\begin{array}{ccc} R\left(\alpha \right)\;R\left(\frac{1+l}{\sqrt{2}}\right)\;Q_{L}\left(l\right)\;C & = & R\left(\beta \right)\;Q_{L}\left(l\right)\;C \end{array}$$

(51) $$\begin{array}{ccc} & = & C\;R\left(\beta \right)\;Q_{R}\left(-l\right) \end{array}$$

(52) $$\begin{array}{ccc} & = & C\;Q_{R}\left(-\beta l\beta^{†}\right)\;R\left(\beta \right) \end{array}$$

(53) $$\begin{array}{ccc} & = & C\;Q_{L}\left(-\beta l\beta^{†}\right)\;R\left(\beta l\right) \end{array}$$

and

(54) $$\begin{array}{ccc} C\;R\left(\alpha \right)\;R\left(\frac{1+l}{\sqrt{2}}\right)\;Q_{L}\left(l\right)\;C & = & C\;R\left(\beta \right)\;Q_{L}\left(l\right)\;C \end{array}$$

(55) $$\begin{array}{ccc} & = & C^{2}\;R\left(\beta \right)\;Q_{R}\left(-l\right) \end{array}$$

(56) $$\begin{array}{ccc} & = & Q_{R}\left(-\beta \;l\;\beta^{†}\right)\;R\left(\beta \right) \end{array}$$

(57) $$\begin{array}{ccc} & = & Q_{L}\left(-\beta \;l\;\beta^{†}\right)\;R\left(\beta \;l\right). \end{array}$$

From $-\beta \;l\;\beta^{†}\in \pm \Lambda$ and βl∈±Ω, the operators of step (B) are represented as $Q_{L}\left(h\right)\;R\left(\alpha \right)$ or $C\;Q_{L}\left(h\right)\;R\left(\alpha \right)$ with h∈±Λ and α∈±Ω. □

Finally, we determine all the permutations of the axes through step (C).

**Proposition** **3.** *The set of all the permutations of the axes is*

(58) $$\begin{array}{ccc} \Gamma & = & \left\{Q_{L}\left(h\right)\;R\left(\alpha \right),\;C\;Q_{L}\left(h\right)\;R\left(\alpha \right)\;;\;h\in \pm \Lambda^{+},\;\alpha \in \pm \Omega \right\}. \end{array}$$

**Proof.**  We note that the operators of steps (A) and (B) are included in Γ. The operator −C can be applied to reverse only the real axis. We apply −C to $Q_{L}\left(h\right)\;R\left(\alpha \right)$ and $Q_{L}\left(h\right)\;R\left(\alpha \right)\;C$. From Lemmas 2, 3 and 5, the following equalities are obtained:

(59) $$\begin{array}{ccc} -C\;Q_{L}\left(h\right)\;R\left(\alpha \right) & = & C\;Q_{L}\left(-h\right)\;R\left(\alpha \right) \end{array}$$

(60) $$\begin{array}{ccc} -C^{2}\;Q_{L}\left(h\right)\;R\left(\alpha \right) & = & -Q_{L}\left(h\right)\;R\left(\alpha \right) \end{array}$$

(61) $$\begin{array}{ccc} & = & Q_{L}\left(-h\right)\;R\left(\alpha \right) \end{array}$$

These belong to Γ again. Inversely, all operators in Γ clearly permute the axes. Thus, all the permutations are determined. □

The number of the permutations of axes is $4!\times 2^{4}=384$, where 4! and $2^{4}$ are the number of axis permutations and direction changes, respectively. This coincides with $2\left|\pm \Lambda \right|\left|\Omega \right|$, where ‘double’ corresponds to whether *C* exists in the operators.

We provide a few examples.

**Example** **4.** *The transformation $C\;R_{k}\left(\pi \right)=C\;R\left(k\right)$ induces that only the k-axis changes the direction:*

(62) $$\begin{array}{ccc} {\left(k\;q(-k)\right)}^{†} & = & q_{0}+q_{1}\;i+q_{2}\;j-q_{3}\;k, \end{array}$$

(63) $$\begin{array}{ccc} C\;R(k) & = & C\;Q_{L}\left(k\right)\;Q_{R}\left(k\right) \end{array}$$

(64) $$\begin{array}{ccc} & = & \left(\begin{array}{cccc} 1 & 0 & 0 & 0\\ 0 & 1 & 0 & 0\\ 0 & 0 & 1 & 0\\ 0 & 0 & 0 & -1 \end{array}\right). \end{array}$$

**Example** **5.** *The transformation $C\;R_{i}\left(\frac{\pi }{2}\right)R_{k}\left(\pi \right)=C\;R\left(\frac{k-j}{\sqrt{2}}\right)$ exchanges the j- and k-axes:*

(65) $$\begin{array}{ccc} {\left(\left(\frac{k-j}{\sqrt{2}}\right)\;q\left(\frac{j-k}{\sqrt{2}}\right)\right)}^{†} & = & \frac{1}{2}\left(k-j\right)\;q^{†}\left(j-k\right) \end{array}$$

(66) $$\begin{array}{ccc} & = & q_{0}+q_{1}i+q_{3}j+q_{2}k, \end{array}$$

(67) $$\begin{array}{ccc} C\;R\left(\frac{k-j}{\sqrt{2}}\right) & = & \frac{1}{2}C\;Q_{L}\left(k-j\right)\;Q_{R}\left(j-k\right) \end{array}$$

(68) $$\begin{array}{ccc} & = & \left(\begin{array}{cccc} 1 & 0 & 0 & 0\\ 0 & 1 & 0 & 0\\ 0 & 0 & 0 & 1\\ 0 & 0 & 1 & 0 \end{array}\right). \end{array}$$

**Example** **6.** *The transformation $R\left(\frac{1+i+j+k}{2}\right)$ rotates the i-, j- and k-axes:*

(69) $$\begin{array}{ccc} \left(\frac{1+i+j+k}{2}\right)\;q\;\left(\frac{1-i-j-k}{2}\right) & = & q_{0}+q_{3}i+q_{1}j+q_{2}k, \end{array}$$

(70) $$\begin{array}{ccc} R\left(\frac{1+i+j+k}{2}\right) & = & \frac{1}{4}\;Q_{L}\left(1+i+j+k\right)\;Q_{R}\left(1-i-j-k\right) \end{array}$$

(71) $$\begin{array}{ccc} & = & \left(\begin{array}{cccc} 1 & 0 & 0 & 0\\ 0 & 0 & 0 & 1\\ 0 & 1 & 0 & 0\\ 0 & 0 & 1 & 0 \end{array}\right). \end{array}$$

## 6. Transformation Equivalence of QVMLPs

The left QVMLP $F_{L}\left(\vec{x};U,\vec{v}\right)$ was implemented using the right QVMLP $F_{R}\left(\vec{x};U^{†},{\vec{v}}^{†}\right)$ with $\phi \left(\vec{x}\right)={\vec{x}}^{\;†}$ and $\psi \left(z\right)=z^{†}$ in [16]. This is the first example of T-equivalent QVMLPs. In this work, we provide a variety of T-equivalent QVMLPs. We provide T-equivalence corresponding to Proposition 3. We note that the activation function and the permutations of axes are commutative. We denote α∈±Ω, $h\in \pm \Lambda^{+}$, and $A\in {\mathrm{GL}}_{m}\left(H\right)$, and note $\alpha^{†}\alpha =h^{†}h=1$. We list the necessary operations for transformations.

(72) $$\begin{array}{ccc} \alpha f\left(q\right)\alpha^{†} & = & f(\alpha \;q\;\alpha^{†}) \end{array}$$

(73) $$\begin{array}{ccc} hf(q) & = & f(hq) \end{array}$$

(74) $$\begin{array}{ccc} f{\left(q\right)}^{†} & = & f(q^{†}) \end{array}$$

(75) $$\begin{array}{ccc} U\vec{x} & = & \left(UA^{-1}\right)\left(A\vec{x}\right) \end{array}$$

We only consider T-equivalent QVMLPs for left QVMLPs. First, we provide T-equivalence corresponding to $Q_{L}\left(h\right)\;R\left(\alpha \right)$.

**Theorem** **1.** *$F_{L}\left(\vec{x}\;;\;U,\vec{v}\right)$ is T-equivalent to $F_{L}\left(\vec{x}\;;\;\alpha UA^{-1},h\;\alpha \;\vec{v}\;\alpha^{†}\right)$ with $\phi \left(\vec{x}\right)=A\vec{x}\alpha^{†}$ and $\psi \left(z\right)=\alpha^{†}h^{†}z\;\alpha$.*

**Proof.**  (76) $$\begin{array}{ccc} h\;\alpha z\alpha^{†} & = & h\;\alpha \;f\left(\vec{v}\cdot f\left(U\vec{x}\right)\right)\alpha^{†} \end{array}$$

(77) $$\begin{array}{ccc} & = & f\left(\left(h\;\alpha \;\vec{v}\;\alpha^{†}\right)\cdot \left(\alpha \;f\left(U\vec{x}\right)\alpha^{†}\right)\right) \end{array}$$

(78) $$\begin{array}{ccc} & = & f\left(\left(h\;\alpha \;\vec{v}\;\alpha^{†}\right)\cdot f\left(\alpha \;U\vec{x}\alpha^{†}\right)\right) \end{array}$$

(79) $$\begin{array}{ccc} & = & f\left(\left(h\;\alpha \;\vec{v}\;\alpha^{†}\right)\cdot f\left(\alpha \;U\vec{x}\alpha^{†}\right)\right) \end{array}$$

(80) $$\begin{array}{ccc} & = & f\left(\left(h\;\alpha \;\vec{v}\;\alpha^{†}\right)\cdot f\left(\left(\alpha \;UA^{-1}\right)\left(A\vec{x}\alpha^{†}\right)\right)\right) \end{array}$$

(81) $$\begin{array}{ccc} & = & F_{L}\left(A\vec{x}\alpha^{†}\;;\;\alpha UA^{-1},h\;\alpha \;\vec{v}\;\alpha^{†}\right) \end{array}$$

From $z=\psi \left(F_{L}\left(A\vec{x}\alpha^{†}\;;\;\alpha UA^{-1},h\;\alpha \;\vec{v}\;\alpha^{†}\right)\right)$, $F_{L}\left(\vec{x}\;;\;U,\vec{v}\right)$ is T-equivalent to $F_{L}\left(\vec{x}\;;\;\alpha UA^{-1},h\;\alpha \;\vec{v}\;\alpha^{†}\right)$ with $\phi \left(\vec{x}\right)=A\vec{x}\alpha^{†}$ and $\psi \left(z\right)=\alpha^{†}h^{†}z\;\alpha$. □

We decompose the transformation used in Theorem 1.

**Figure 9:**
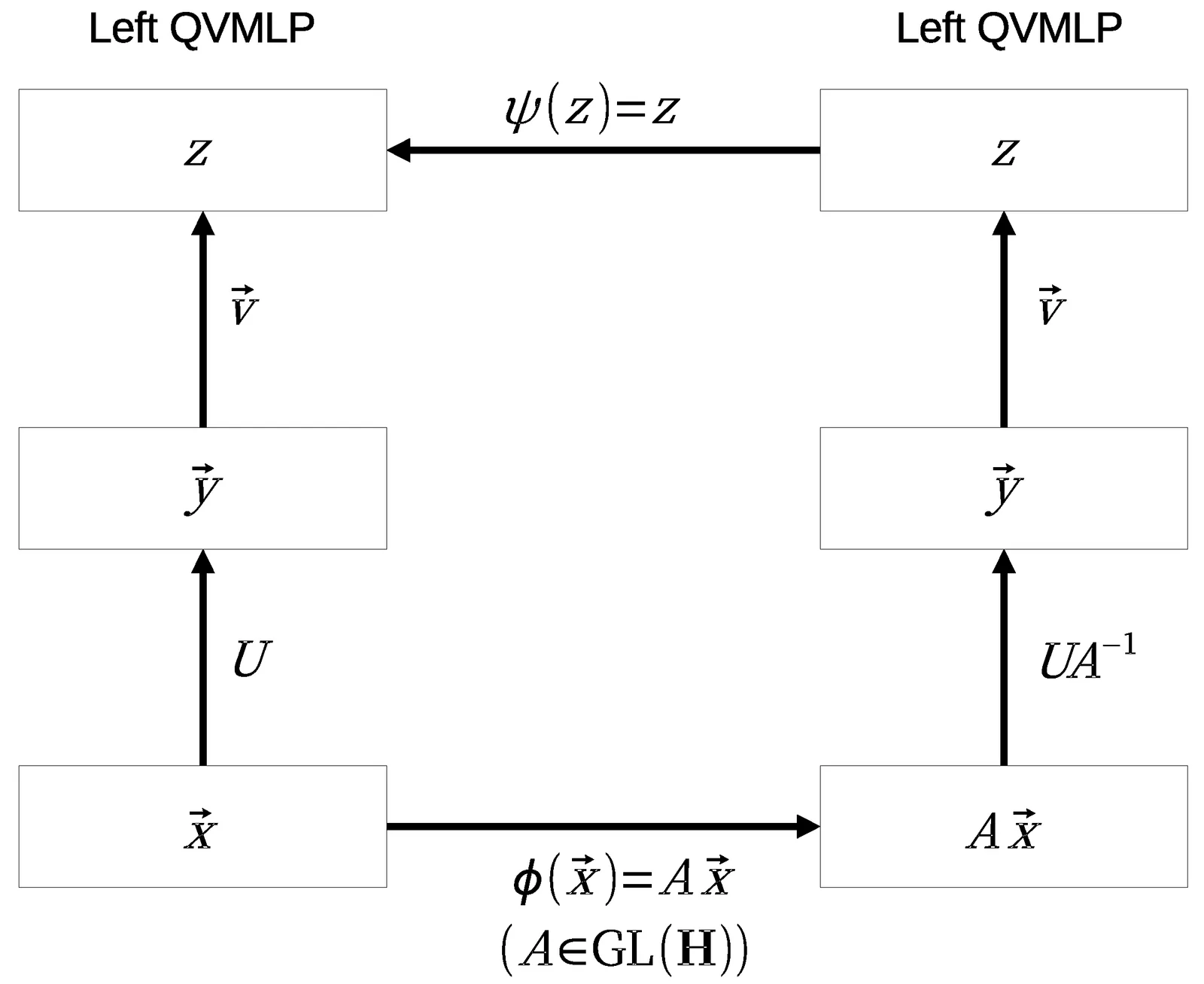
$F_{L}\left(\vec{x}\;;\;U,\vec{v}\;\right)\mapsto F_{L}\left(\vec{x}\;;\;UA^{-1},\vec{v}\;\right)$ with $\phi \left(\vec{x}\right)=A\vec{x}$ and ψ(z)=z.

**Figure 10:**
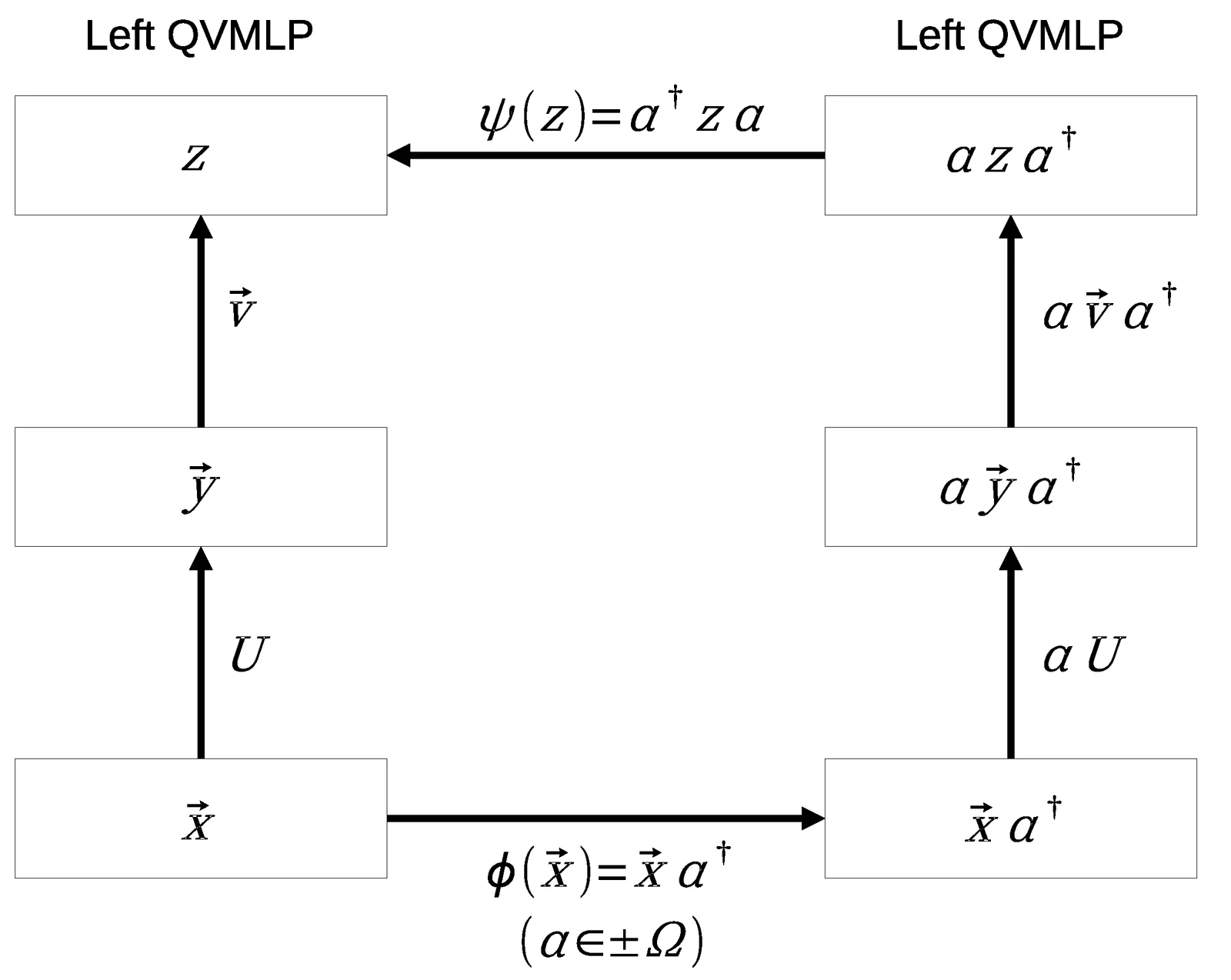
$F_{L}\left(\vec{x}\;;\;U,\vec{v}\;\right)\mapsto F_{L}\left(\vec{x}\;;\;\alpha U,\alpha \vec{v}\alpha^{†}\right)$ with $\phi \left(\vec{x}\right)=\vec{x}\alpha^{†}$ and $\psi \left(z\right)=\alpha^{†}z\alpha$.

**Figure 11:**
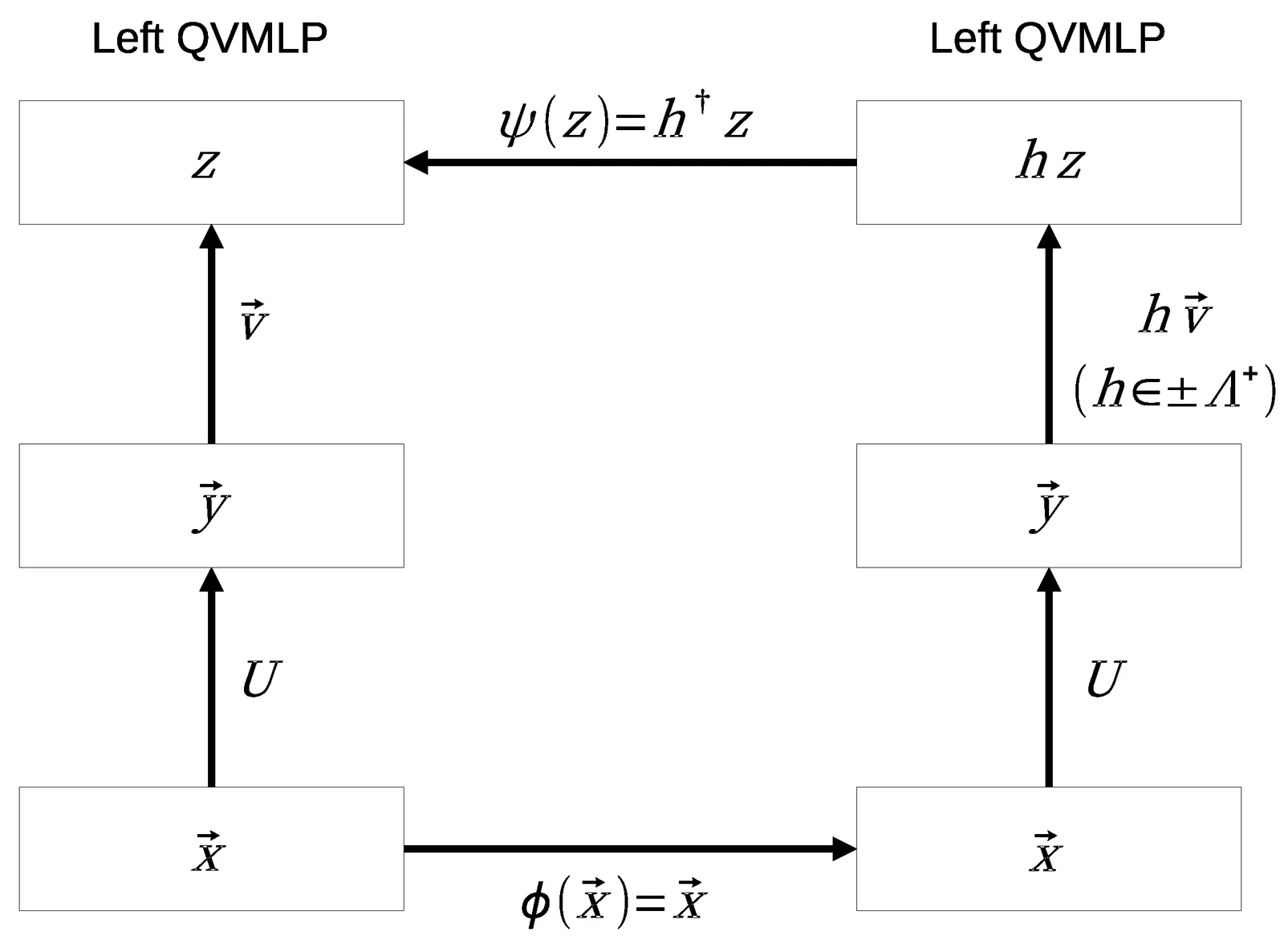
$F_{L}\left(\vec{x}\;;\;U,\vec{v}\;\right)\mapsto F_{L}\left(\vec{x}\;;\;U,h\vec{v}\;\right)$ with $\phi \left(\vec{x}\right)=\vec{x}$ and $\psi \left(z\right)=h^{†}z$.

- $F_{L}\left(\vec{x}\;;\;U,\vec{v}\;\right)\mapsto F_{L}\left(\vec{x}\;;\;UA^{-1},\vec{v}\;\right)$ with $\phi \left(\vec{x}\right)=A\vec{x}$ and ψ(z)=z (Figure 9).
- $F_{L}\left(\vec{x}\;;\;U,\vec{v}\;\right)\mapsto F_{L}\left(\vec{x}\;;\;\alpha U,\alpha \vec{v}\alpha^{†}\right)$ with $\phi \left(\vec{x}\right)=\vec{x}\alpha^{†}$ and $\psi \left(z\right)=\alpha^{†}z\alpha$ (Figure 10).
- $F_{L}\left(\vec{x}\;;\;U,\vec{v}\;\right)\mapsto F_{L}\left(\vec{x}\;;\;U,h\vec{v}\;\right)$ with $\phi \left(\vec{x}\right)=\vec{x}$ and $\psi \left(z\right)=h^{†}z$ (Figure 11).

In fact, we obtain the following sequence of translations from these operations.

(82) $$\begin{array}{ccc} F_{L}\left(\vec{x}\;;\;U,\vec{v}\right) & \mapsto & F_{L}\left(\vec{x}\;;\;UA^{-1},\vec{v}\right)\;\;\mathrm{with}\;\;\phi \left(\vec{x}\right)=A\vec{x}\;\;\mathrm{and}\;\;\psi \left(z\right)=z \end{array}$$

(83) $$\begin{array}{ccc} & \mapsto & F_{L}\left(\vec{x}\;;\;\alpha UA^{-1},\alpha \vec{v}\alpha^{†}\right)\\ & & \mathrm{with}\;\;\phi \left(\vec{x}\right)=\left(A\vec{x}\right)\alpha^{†}=A\vec{x}\alpha^{†}\;\;\mathrm{and}\;\;\psi \left(z\right)=\alpha^{†}z\alpha \end{array}$$

(84) $$\begin{array}{ccc} & \mapsto & F_{L}\left(\vec{x}\;;\;\alpha UA^{-1},h\alpha \vec{v}\alpha^{†}\right)\\ & & \mathrm{with}\;\;\phi \left(\vec{x}\right)=A\vec{x}\alpha^{†}\;\;\mathrm{and}\;\;\psi \left(z\right)=\alpha^{†}\left(h^{†}z\right)\alpha =\alpha^{†}h^{†}z\alpha \end{array}$$

Next, we consider the T-equivalence corresponding to $C\;Q_{L}\left(h\right)\;R\left(\alpha \right)$.

**Theorem** **2.** *$F_{L}\left(\vec{x}\;;\;U,\vec{v}\right)$ is T-equivalent to $F_{R}\left(\vec{x}\;;\;{\left(UA^{-1}\right)}^{†}\alpha^{†},\alpha \;{\vec{v}}^{\;†}\alpha^{†}h^{†}\right)$ with $\phi \left(\vec{x}\right)=\alpha {\left(A\vec{x}\right)}^{†}$ and $\psi \left(z\right)=\alpha^{†}h^{†}z^{†}\alpha$. In particular, there exist right QVMLPs which are T-equivalent to a given left QVMLP.*

**Proof.**  We apply opetator *C* after $Q_{L}\left(h\right)\;R\left(\alpha \right)$. First, we determine the transformation using *C*.

(85) $$\begin{array}{ccc} z^{†} & = & f{\left(\vec{v}\cdot f\left(U\vec{x}\right)\right)}^{†} \end{array}$$

(86) $$\begin{array}{ccc} & = & f\left(f{\left(U\vec{x}\right)}^{†}\cdot {\vec{v}}^{†}\right) \end{array}$$

(87) $$\begin{array}{ccc} & = & f\left(f{\left(U\vec{x}\right)}^{H}\;{\vec{v}}^{†}\right) \end{array}$$

(88) $$\begin{array}{ccc} & = & f\left(f\left({\vec{x}}^{H}U^{H}\right)\;{\vec{v}}^{†}\right) \end{array}$$

(89) $$\begin{array}{ccc} & = & F_{R}\left({\vec{x}}^{\;†}\;;\;U^{†},{\vec{v}}^{\;†}\right) \end{array}$$

The transformation corresponding to *C* is

(90) $$\begin{array}{ccc} F_{L}\left(\vec{x}\;;\;U,\vec{v}\right) & \mapsto & F_{R}\left(\vec{x}\;;\;U^{†},{\vec{v}}^{\;†}\right)\;\;\mathrm{with}\;\;\phi \left(\vec{x}\right)={\vec{x}}^{\;†}\;\;\mathrm{and}\;\;\psi \left(z\right)=z^{†}. \end{array}$$

Next, we apply operator *C* to (84).

(91) $$\begin{array}{ccc} & & F_{L}\left(\vec{x}\;;\;\alpha UA^{-1},h\alpha \vec{v}\alpha^{†}\right)\\ & & \mathrm{with}\;\;\phi \left(\vec{x}\right)=A\vec{x}\alpha^{†}\;\;\mathrm{and}\;\;\psi \left(z\right)=\alpha^{†}\left(h^{†}z\right)\alpha =\alpha^{†}h^{†}z\alpha \end{array}$$

(92) $$\begin{array}{ccc} & \mapsto & F_{R}\left(\vec{x}\;;\;{\left(UA^{-1}\right)}^{†}\alpha^{†},\alpha {\vec{v}}^{\;†}\alpha^{†}h^{†}\right)\\ & & \mathrm{with}\;\;\phi \left(\vec{x}\right)=\alpha {\left(A\vec{x}\right)}^{†}\;\;\mathrm{and}\;\;\psi \left(z\right)=\alpha^{†}h^{†}z^{†}\alpha \end{array}$$

□

**Figure 12:**
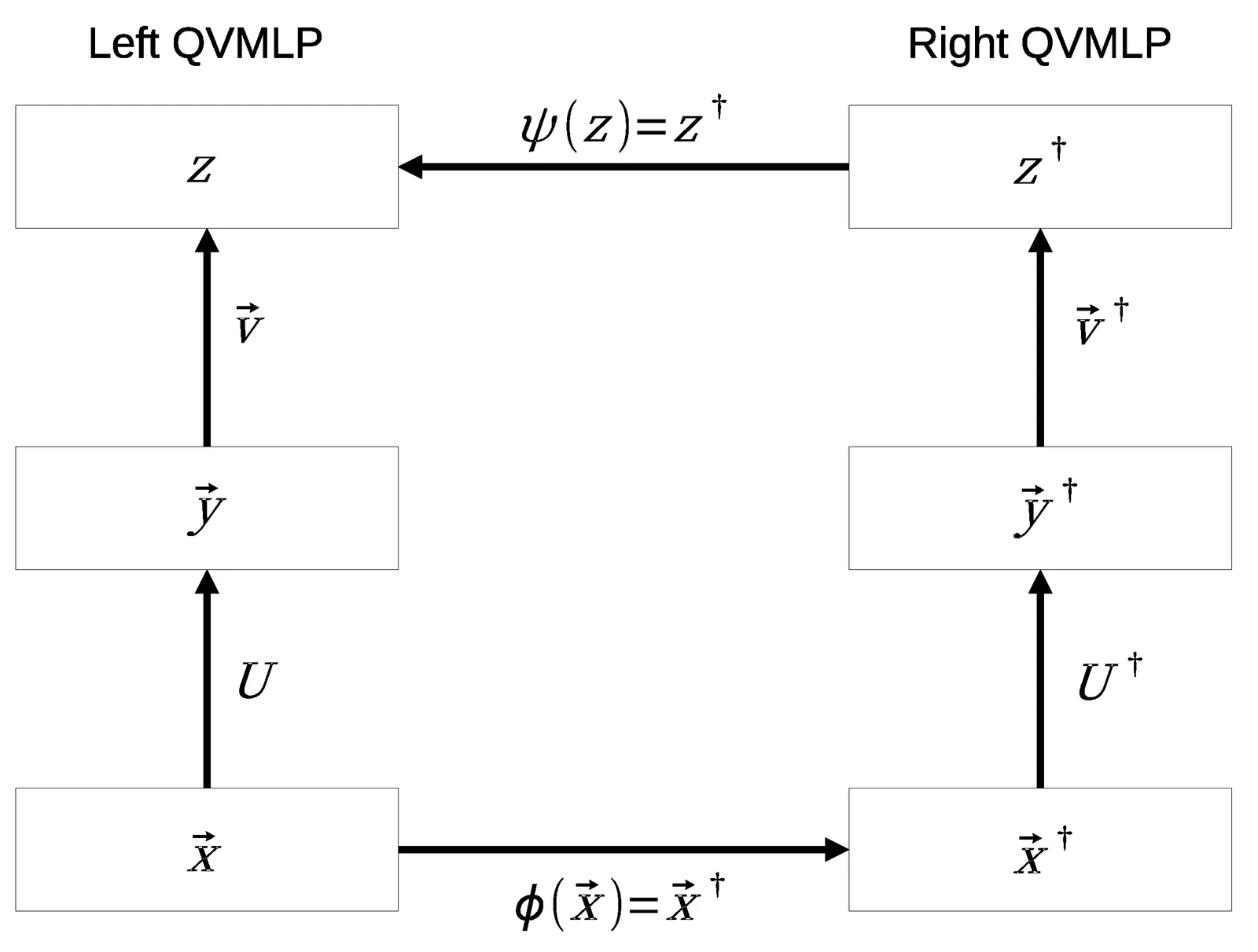
$F_{L}\left(\vec{x}\;;\;U,\vec{v}\right)$ is T-equivalent to $F_{R}\left(\vec{x}\;;\;U^{†},{\vec{v}}^{\;†}\right)$ with $\phi \left(\vec{x}\right)={\vec{x}}^{\;†}$ and $\psi \left(z\right)=z^{†}$.

**Example** **7.** *$F_{L}\left(\vec{x}\;;\;U,\vec{v}\right)$ is T-equivalent to $F_{R}\left(\vec{x}\;;\;U^{†},{\vec{v}}^{\;†}\right)$ with $\phi \left(\vec{x}\right)={\vec{x}}^{\;†}$ and $\psi \left(z\right)=z^{†}$ (Figure 12).*

We confirm Theorem 2 through direct calculation.

(93) $$\begin{array}{ccc} F_{R}\left(\phi \left(\vec{x}\right)\;;\;{\left(UA^{-1}\right)}^{†}\alpha^{†},\alpha \;{\vec{v}}^{\;†}\alpha^{†}h^{†}\right) & = & f\left(f\left({\left(\alpha {\left(A\vec{x}\right)}^{†}\right)}^{T}{\left({\left(UA^{-1}\right)}^{†}\alpha^{†}\right)}^{T}\right)\;\alpha \;{\vec{v}}^{\;†}\alpha^{†}h^{†}\right) \end{array}$$

(94) $$\begin{array}{ccc} & = & f\left(f\left(\alpha {\left(A\vec{x}\right)}^{H}{\left(UA^{-1}\right)}^{H}\alpha^{†}\right)\;\alpha \;{\vec{v}}^{\;†}\alpha^{†}\right)h^{†} \end{array}$$

(95) $$\begin{array}{ccc} & = & f\left(\alpha f\left({\vec{x}}^{\;H}U^{H}\right)\;{\vec{v}}^{\;†}\alpha^{†}\right)h^{†} \end{array}$$

(96) $$\begin{array}{ccc} & = & \alpha f\left(f\left({\left(U\vec{x}\right)}^{H}\right)\;{\vec{v}}^{\;†}\right)\alpha^{†}h^{†} \end{array}$$

(97) $$\begin{array}{ccc} & = & \alpha f\left(f{\left(U\vec{x}\right)}^{\;†}\cdot \;{\vec{v}}^{\;†}\right)\alpha^{†}h^{†} \end{array}$$

(98) $$\begin{array}{ccc} & = & \alpha f{\left(\vec{v}\cdot f\left(U\vec{x}\right)\right)}^{\;†}\alpha^{†}h^{†} \end{array}$$

(99) $$\begin{array}{ccc} & = & \alpha F_{L}{\left(\vec{x}\;;\;U,\vec{v}\right)}^{†}\alpha^{†}h^{†} \end{array}$$

(100) $$\begin{array}{ccc} \psi \left(\alpha F_{L}{\left(\vec{x}\;;\;U,\vec{v}\right)}^{†}\alpha^{†}h^{†}\right) & = & \alpha^{†}h^{†}{\left(\alpha F_{L}{\left(\vec{x}\;;\;U,\vec{v}\right)}^{†}\alpha^{†}h^{†}\right)}^{†}\alpha \end{array}$$

(101) $$\begin{array}{ccc} & = & F_{L}\left(\vec{x}\;;\;U,\vec{v}\right) \end{array}$$

We provide the algorithm to obtain T-equivalent QVMLPs of a given minimal left QVMLP and an example.

1. We transform the given left QVMLP to an I/O-equivalent QVMLP using the operations described in Section 3.
  (102) $$\left(U,\vec{v}\right)\mapsto \left(DPU,{\left({\left(P\vec{v}\right)}^{T}D^{†}\right)}^{T}\right)$$
2. We transform the left QVMLP obtained in step 1 to a T-equivalent left QVMLP.
  (103) $$\left(U,\vec{v}\right)\mapsto \left(\alpha UA^{-1},h\alpha \vec{v}\alpha^{†}\right)\;\mathrm{with}\;\phi \left(\vec{x}\right)=A\vec{x}\alpha^{†}\;\mathrm{and}\;\psi \left(z\right)=\alpha^{†}h^{†}z\alpha$$
3. We transform the left QVMLP obtained in step 2 to a T-equivalent right QVMLP.
  (104) $$\left(U,\vec{v}\right)\mapsto \left(U^{†},{\vec{v}}^{\;†}\right)\;\mathrm{with}\;\phi \left(\vec{x}\right)={\vec{x}}^{\;†}\;\mathrm{and}\;\psi \left(z\right)=z^{†}$$

**Example** **8.** *The left QVMLP $F_{L}\left(\vec{x}\;;\;U,\vec{v}\right)$ with $U=\left(\begin{array}{cc} 1 & i\\ j & k \end{array}\right)$ and $\vec{v}=\left(\begin{array}{c} 1\\ i \end{array}\right)$ is a minimal left QVMLP with m=n=2. We apply the above three steps to*

(105) $$\begin{array}{ccc} F_{L}\left(\vec{x}\;;\;U,\vec{v}\right) & = & f\left(\;f\left(x_{1}+ix_{2}\right)+i\;f\left(jx_{1}+kx_{2}\right)\;\right) \end{array}$$

(106) $$\begin{array}{ccc} & = & f\left(\;f\left(x_{1}+ix_{2}\right)+f\left(kx_{1}-jx_{2}\right)\;\right). \end{array}$$

*1.*  *We take $P=\left(\begin{array}{cc} 0 & 1\\ 1 & 0 \end{array}\right)$ and $D=\left(\begin{array}{cc} i & 0\\ 0 & -j \end{array}\right)$. Then, $F_{L}\left(\vec{x}\;;\;U,\vec{v}\right)$ is I/O-equivalent to $F_{L}\left(\vec{x}\;;\;U^{\prime },\vec{v^{\prime }}\right)$;*
(107) $$\begin{array}{ccc} U^{\prime } & = & DPU\;=\;\left(\begin{array}{cc} i & 0\\ 0 & -j \end{array}\right)\left(\begin{array}{cc} 0 & 1\\ 1 & 0 \end{array}\right)\left(\begin{array}{cc} 1 & i\\ j & k \end{array}\right)\;=\;\left(\begin{array}{cc} k & -j\\ -j & k \end{array}\right), \end{array}$$
(108) $$\begin{array}{ccc} \vec{v^{\prime }} & = & {\left({\left(P\vec{v}\right)}^{T}D^{†}\right)}^{T}\;=\;{\left({\left(\left(\begin{array}{cc} 0 & 1\\ 1 & 0 \end{array}\right)\left(\begin{array}{c} 1\\ i \end{array}\right)\right)}^{T}\left(\begin{array}{cc} -i & 0\\ 0 & j \end{array}\right)\right)}^{T} \end{array}$$
(109) $$\begin{array}{ccc} & = & {\left(\left(\begin{array}{cc} i & 1 \end{array}\right)\left(\begin{array}{cc} -i & 0\\ 0 & j \end{array}\right)\right)}^{T}\;=\;\left(\begin{array}{c} 1\\ j \end{array}\right). \end{array}$$
*In fact, we obtain*
(110) $$\begin{array}{ccc} F_{L}\left(\vec{x}\;;\;U^{\prime },\vec{v^{\prime }}\right) & = & f\left(\;f\left(kx_{1}-jx_{2}\right)+j\;f\left(-jx_{1}+kx_{2}\right)\;\right) \end{array}$$
(111) $$\begin{array}{ccc} & = & f\left(\;f\left(kx_{1}-jx_{2}\right)+f\left(x_{1}+ix_{2}\right)\;\right) \end{array}$$
(112) $$\begin{array}{ccc} & = & F_{L}\left(\vec{x}\;;\;U,\vec{v}\right). \end{array}$$
*2.*  *We take A=I, $\alpha =\frac{j-k}{\sqrt{2}}$, and h=i. Then, $F_{L}\left(\vec{x}\;;\;U,\vec{v}\right)$ is T-equivalent to $F_{L}\left(\vec{x}\;;\;U^{\prime \prime },\vec{v^{\prime \prime }}\right)$:*
(113) $$\begin{array}{ccc} U^{\prime \prime } & = & \alpha U^{\prime }A^{-1}\;=\;\frac{j-k}{\sqrt{2}}\left(\begin{array}{cc} k & -j\\ -j & k \end{array}\right)\;=\;\frac{1}{\sqrt{2}}\left(\begin{array}{cc} 1+i & 1-i\\ 1-i & 1+i \end{array}\right), \end{array}$$
(114) $$\begin{array}{ccc} \vec{v^{\prime \prime }} & = & h\alpha \vec{v^{\prime }}\alpha^{†}\;=\;\frac{i\;(j-k)}{\sqrt{2}}\left(\begin{array}{c} 1\\ j \end{array}\right)\frac{k-j}{\sqrt{2}}\;=\;\left(\begin{array}{c} i\\ j \end{array}\right), \end{array}$$
(115) $$\begin{array}{ccc} \phi (\vec{x}) & = & \left(\begin{array}{c} x_{1}\\ x_{2} \end{array}\right)\;\frac{k-j}{\sqrt{2}}, \end{array}$$
(116) $$\begin{array}{ccc} \psi (z) & = & \alpha^{†}h^{†}z\alpha \;=\;\frac{\left(k-j)(-i\right)}{\sqrt{2}}\;z\;\frac{j-k}{\sqrt{2}}\;=\;\frac{j+k}{\sqrt{2}}\;z\;\frac{k-j}{\sqrt{2}}. \end{array}$$
*In fact, we obtain*
$$\begin{array}{ccc} & & \psi \left(F_{L}\left(\phi \left(\vec{x}\right)\;;\;U^{\prime \prime },\vec{v^{\prime \prime }}\right)\;\right) \end{array}$$
(117) $$\begin{array}{ccc} & = & \frac{\left(k-j)(-i\right)}{\sqrt{2}}\;f\left(i\;f\left(\left(\frac{1+i}{\sqrt{2}}x_{1}+\frac{1-i}{\sqrt{2}}x_{2}\right)\;\frac{k-j}{\sqrt{2}}\right)+j\;f\left(\left(\frac{1-i}{\sqrt{2}}x_{1}+\frac{1+i}{\sqrt{2}}x_{2}\right)\;\frac{k-j}{\sqrt{2}}\right)\;\right)\;\frac{j-k}{\sqrt{2}} \end{array}$$
(118) $$\begin{array}{ccc} & = & \frac{k-j}{\sqrt{2}}\;f\left(\;f\left(\left(\frac{1+i}{\sqrt{2}}x_{1}+\frac{1-i}{\sqrt{2}}x_{2}\right)\;\frac{k-j}{\sqrt{2}}\right)-k\;f\left(\left(\frac{1-i}{\sqrt{2}}x_{1}+\frac{1+i}{\sqrt{2}}x_{2}\;\right)\;\frac{k-j}{\sqrt{2}}\right)\;\right)\;\frac{j-k}{\sqrt{2}} \end{array}$$
(119) $$\begin{array}{ccc} & = & \frac{k-j}{\sqrt{2}}\;f\left(\;f\left(\left(\frac{1+i}{\sqrt{2}}x_{1}+\frac{1-i}{\sqrt{2}}x_{2}\right)\;\frac{k-j}{\sqrt{2}}\right)+\;f\left(\left(\frac{j-k}{\sqrt{2}}x_{1}-\frac{j+k}{\sqrt{2}}x_{2}\right)\;\frac{k-j}{\sqrt{2}}\right)\;\right)\;\frac{j-k}{\sqrt{2}} \end{array}$$
(120) $$\begin{array}{ccc} & = & f\left(\;f\left(\frac{k-j}{\sqrt{2}}\;\left(\frac{1+i}{\sqrt{2}}x_{1}+\frac{1-i}{\sqrt{2}}x_{2}\right)\;\frac{k-j}{\sqrt{2}}\;\frac{j-k}{\sqrt{2}}\right)+\;f\left(\frac{k-j}{\sqrt{2}}\;\left(\frac{j-k}{\sqrt{2}}x_{1}-\frac{j+k}{\sqrt{2}}x_{2}\right)\;\frac{k-j}{\sqrt{2}}\;\frac{j-k}{\sqrt{2}}\right)\;\right) \end{array}$$
(121) $$\begin{array}{ccc} & = & f\left(\;f\left(\frac{k-j}{\sqrt{2}}\;\left(\frac{1+i}{\sqrt{2}}x_{1}+\frac{1-i}{\sqrt{2}}x_{2}\right)\right)+\;f\left(\frac{k-j}{\sqrt{2}}\;\left(\frac{j-k}{\sqrt{2}}x_{1}-\frac{j+k}{\sqrt{2}}x_{2}\right)\right)\;\right) \end{array}$$
(122) $$\begin{array}{ccc} & = & f\left(\;f\left(kx_{1}-jx_{2}\right)+f\left(x_{1}+ix_{2}\right)\;\right) \end{array}$$
(123) $$\begin{array}{ccc} & = & F_{L}\left(\vec{x}\;;\;U,\vec{v}\right). \end{array}$$
*3.*  *$F_{L}\left(\vec{x}\;;\;U,\vec{v}\right)$ is T-equivalent to $F_{R}\left(\vec{x}\;;\;U^{\prime \prime †},{\vec{v^{\prime \prime }}}^{\;†}\right)$:*
(124) $$\begin{array}{ccc} U^{\prime \prime †} & = & \frac{1}{\sqrt{2}}{\left(\begin{array}{cc} 1+i & 1-i\\ 1-i & 1+i \end{array}\right)}^{†}\;=\;\frac{1}{\sqrt{2}}\left(\begin{array}{cc} 1-i & 1+i\\ 1+i & 1-i \end{array}\right), \end{array}$$
(125) $$\begin{array}{ccc} {\vec{v^{\prime \prime }}}^{\;†} & = & {\left(\begin{array}{c} i\\ j \end{array}\right)}^{†}\;=\;\left(\begin{array}{c} -i\\ -j \end{array}\right), \end{array}$$
(126) $$\begin{array}{ccc} \phi (\vec{x}) & = & {\left(\left(\begin{array}{c} x_{1}\\ x_{2} \end{array}\right)\;\frac{k-j}{\sqrt{2}}\right)}^{†}\;=\;\frac{j-k}{\sqrt{2}}\left(\begin{array}{c} x_{1}^{†}\\ x_{2}^{†} \end{array}\right), \end{array}$$
(127) $$\begin{array}{ccc} \psi (z) & = & \frac{\left(k-j)(-i\right)}{\sqrt{2}}\;z^{†}\;\frac{j-k}{\sqrt{2}}\;=\;\frac{j+k}{\sqrt{2}}\;z^{†}\;\frac{k-j}{\sqrt{2}}. \end{array}$$
*In fact, we obtain*
$$\begin{array}{ccc} & & \psi \left(\;F_{R}\left(\phi \left(\vec{x}\right)\;;\;U^{\prime \prime †},{\vec{v^{\prime \prime }}}^{\;†}\right)\;\right) \end{array}$$
(128) $$\begin{array}{ccc} & = & \frac{\left(k-j)(-i\right)}{\sqrt{2}}\;f\left(\;f\left(\frac{j-k}{\sqrt{2}}\;x_{1}^{†}\;\frac{1-i}{\sqrt{2}}+\frac{j-k}{\sqrt{2}}\;x_{2}^{†}\;\frac{1+i}{\sqrt{2}}\right)\;\left(-i\right)\right.\\ & & \;\;\;\;\;\;\;{\left.+f\left(\frac{j-k}{\sqrt{2}}\;x_{1}^{†}\;\frac{1+i}{\sqrt{2}}+\frac{j-k}{\sqrt{2}}\;x_{2}^{†}\;\frac{1-i}{\sqrt{2}}\right)\;\left(-j\right)\;\right)}^{†}\;\frac{j-k}{\sqrt{2}} \end{array}$$
(129) $$\begin{array}{ccc} & = & \frac{\left(k-j)(-i\right)}{\sqrt{2}}\;f\left(\;f\left(\frac{j-k}{\sqrt{2}}\;x_{1}^{†}\;\frac{-1-i}{\sqrt{2}}+\frac{j-k}{\sqrt{2}}\;x_{2}^{†}\;\frac{1-i}{\sqrt{2}}\right)\right.\\ & & \;\;\;\;\;\;\;{\left.+f\left(\frac{j-k}{\sqrt{2}}\;x_{1}^{†}\;\frac{-j-k}{\sqrt{2}}+\frac{j-k}{\sqrt{2}}x_{2}^{†}\;\frac{k-j}{\sqrt{2}}\right)\;\right)}^{†}\;\frac{j-k}{\sqrt{2}} \end{array}$$
(130) $$\begin{array}{ccc} & = & \frac{\left(k-j)(-i\right)}{\sqrt{2}}\;f\left(\;f\left(\frac{i-1}{\sqrt{2}}\;x_{1}\;\frac{k-j}{\sqrt{2}}+\frac{i+1}{\sqrt{2}}\;x_{2}\;\frac{k-j}{\sqrt{2}}\right)\right.\\ & & \;\;\;\;\;\;\;\left.+f\left(\frac{j+k}{\sqrt{2}}\;x_{1}\;\frac{k-j}{\sqrt{2}}+\frac{j-k}{\sqrt{2}}\;x_{2}\;\frac{k-j}{\sqrt{2}}\right)\;\right)\;\frac{j-k}{\sqrt{2}} \end{array}$$
(131) $$\begin{array}{ccc} & = & \frac{k-j}{\sqrt{2}}\;f\left(\;f\left(\frac{1+i}{\sqrt{2}}\;x_{1}\;\frac{k-j}{\sqrt{2}}+\frac{1-i}{\sqrt{2}}\;x_{2}\;\frac{k-j}{\sqrt{2}}\right)\right.\\ & & \;\;\;\;\;\;\;\left.+f\left(\frac{j-k}{\sqrt{2}}\;x_{1}\;\frac{k-j}{\sqrt{2}}-\frac{j+k}{\sqrt{2}}\;x_{2}\;\frac{k-j}{\sqrt{2}}\right)\;\right)\;\frac{j-k}{\sqrt{2}} \end{array}$$
(132) $$\begin{array}{ccc} & = & f\left(\;f\left(\frac{k-j}{\sqrt{2}}\;\frac{1+i}{\sqrt{2}}\;x_{1}+\frac{k-j}{\sqrt{2}}\;\frac{1-i}{\sqrt{2}}\;x_{2}\right)\right.\\ & & \;\;\;\;\;\;\;\left.+f\left(\frac{k-j}{\sqrt{2}}\;\frac{j-k}{\sqrt{2}}\;x_{1}-\frac{k-j}{\sqrt{2}}\;\frac{j+k}{\sqrt{2}}\;x_{2}\right)\;\right) \end{array}$$
(133) $$\begin{array}{ccc} & = & f\left(\;f\left(k\;x_{1}-j\;x_{2}\right)+f\left(x_{1}+i\;x_{2}\right)\;\right) \end{array}$$
(134) $$\begin{array}{ccc} & = & F_{L}\left(\vec{x}\;;\;U,\vec{v}\right). \end{array}$$

We determine the number of T-equivalent QVMLPs obtained in these steps. We suppose A=I, since the number of *A* belonging to GL(m,H) is infinite. The number of I/O-equivalent left QVMLPs obtained in step 1 has already been determined to be $2^{n}\;n!$ in [15]. The number of T-equivalent left QVMLPs obtained in step 2 is |±Λ||±Ω|=384 for each I/O-equivalent left QVMLP. However, the same QVMLP may be obtained from different I/O-equivalent QVMLPs in step 2. Therefore, at most, $384\times 2^{n}\;n!$ T-equivalent QVMLPs are obtained. We obtain a unique right QVMLP for each left QVMLP in step 3.

**Example** **9.** *$F_{L}\left(\vec{x}\;;\;U,\vec{v}\right)$ and $F_{L}\left(\vec{x}\;;\;-U,-\vec{v}\right)$ are I/O-equivalent. $F_{L}\left(\vec{x}\;;\;U,\vec{v}\right)$ is T-equivalent to $F_{L}\left(\vec{x}\;;\;-U,\vec{v}\right)$ with $\phi \left(\vec{x}\right)=-\vec{x}$ and ψ(z)=z by taking α=−1 and h=1, whereas $F_{L}\left(\vec{x}\;;\;-U,-\vec{v}\right)$ is T-equivalent to $F_{L}\left(\vec{x}\;;\;-U,\vec{v}\right)$ with $\phi \left(\vec{x}\right)=\vec{x}$ and ψ(z)=−z by taking α=1 and h=−1. Although ϕ and ψ are different, the same T-equivalent QVMLP is obtained.*

We summarize the transformation provided in the present work. The transformations are obtained through combinations of the following operations.

(RVMLPs)

- $\left(U,\vec{v}\right)\mapsto \left(UA^{-1},\vec{v}\right)$ with $\phi \left(\vec{x}\right)=A\vec{x}$ and ψ(z)=z (A∈GL(R)). I/O-equivalent if A=I.
- $\left(U,\vec{v}\right)\mapsto \left(\rho_{1}U,\rho_{2}\vec{v}\right)$ with $\phi \left(\vec{x}\right)=\vec{x}$ and $\psi \left(z\right)=\rho_{1}\rho_{2}\;z$ ($\rho_{1},\rho_{2}\in \left\{\pm 1\right\}$). I/O-equivalent if $\rho_{1}\rho_{2}=1$.

(CVMLPs)

- $\left(U,\vec{v}\right)\mapsto \left(UA^{-1},\vec{v}\right)$ with $\phi \left(\vec{x}\right)=A\vec{x}$ and ψ(z)=z (A∈GL(C)). I/O-equivalent if A=I.
- $\left(U,\vec{v}\right)\mapsto \left(\rho_{1}U,\rho_{2}\vec{v}\right)$ with $\phi \left(\vec{x}\right)=\vec{x}$ and $\psi \left(z\right)={\left(\rho_{1}\rho_{2}\right)}^{†}z$ ($\rho_{1},\rho_{2}=\left\{\pm 1,\pm i\right\}$). I/O-equivalent if $\rho_{1}\rho_{2}=1$.
- $\left(U,\vec{v}\right)\mapsto \left(U^{†},{\vec{v}}^{\;†}\right)$ with $\phi \left(\vec{x}\right)={\vec{x}}^{\;†}$ and $\psi \left(z\right)=z^{†}$.

(Left QVMLPs)

- $\left(U,\vec{v}\right)\mapsto \left(UA^{-1},\vec{v}\right)$ with $\phi \left(\vec{x}\right)=A\vec{x}$ and ψ(z)=z (A∈GL(H)). I/O-equivalent if A=I.
- $\left(U,\vec{v}\right)\mapsto \left(\alpha U,\alpha \vec{v}\alpha^{†}\right)$ with $\phi \left(\vec{x}\right)=\vec{x}\alpha^{†}$ and $\psi \left(z\right)=\alpha^{†}z\alpha$ (α∈±Ω). I/O-equivalent if α=1.
- $\left(U,\vec{v}\right)\mapsto \left(U,h\vec{v}\right)$ with $\phi \left(\vec{x}\right)=\vec{x}$ and $\psi \left(z\right)=h^{†}z$ ($h\in \pm \Lambda^{+}$). I/O-equivalent if h=1.
- $\left(U,\vec{v}\right)\mapsto \left(U^{†},{\vec{v}}^{\;†}\right)$ with $\phi \left(\vec{x}\right)={\vec{x}}^{\;†}$ and $\psi \left(z\right)=z^{†}$. The type of QVMLP changes from left to right.

As the dimension of algebra increases, more various transformations are found.

## 7. Conclusions

I/O-equivalence is an important concept for understanding information processing by MLPs. In this paper, I/O-equivalence was extended to T-equivalence. T-equivalence is when two MLPs provide the same results through a transformation of input and output and are considered to provide the same information processing. The T-equivalence of RVMLPs is simple. In the T-equivalence of CVMLPs, conjugate transformations emerge. The T-equivalence of QVMLPs is complicated, since the multiplication of quaternions is not commutative. We investigated the permutations of axes in 4D space and applied them to transformations of QVMLPs. Thus, we found that the conjugate operator changed the types of QVMLPs. We have not determined all the T-equivalent MLPs yet. Determining all the T-equivalent MLPs remains an important problem. The maps ϕ and ψ are required only to be invertible. It might be better to modify the requirement for further studies. In particular, it would be useful if the same operation is independently applied to components of the input vector. Moreover, we can consider further restrictions on operations. For example, in the case of quaternion-valued neural networks, the operations are limited to $x_{a}\mapsto \rho x_{a}$, $x_{a}\mapsto \rho x_{a}^{†}$$\left(\rho \in \pm \Lambda^{+}\right)$, and so on. Although only MLPs with split activation functions are investigated in this paper, those with phasor activation functions should also be studied [19]. The targets of T-equivalence are not limited to MLPs, and this concept is applicable to other learning machines. In particular, we are planning to apply our theory to other models of neural networks, such as Hopfield networks [16].

## Data Availability

Data are contained within the article.

## Abbreviations

The following abbreviations are used in this manuscript:

MLP	Multilayer perceptron
RVMLP	Real-valued multilayer perceptron
CVMLP	Complex-valued multilayer perceptron
QVMLP	Quaternion-valued multilayer perceptron
T-equivalence	Transformation equivalence
